# Supplementary material for: How deep is your art: An experimental study on the limits of artistic understanding in a single-task, single-modality neural network
Source: PLoS One. 2024 Nov 6;19(11):e0305943. doi: 10.1371/journal.pone.0305943 (PMC11540182; doi:10.1371/journal.pone.0305943)
Supplement: S1 Table — (PDF) [file pone.0305943.s001.pdf]

Table 1. List of common terms in art history and visual arts to describe the EXPs

Medium	Color		Shape, Form, Texture		
- Black and white photography<br>- Color photography<br>- Mix media, using photography	- High Saturation<br>- Medium saturation<br>- Low saturation<br>- Cool colors<br>- Warm colors<br>- Neutral colors<br>- Dark Value<br>- Light value	- Mid-tones<br>- High Contrast<br>- Low contrast<br>- Medium contrast<br>- Monochromatic<br>- Chromatic<br>- Colorful	- Figurative<br>- Organic<br>- Geometric<br>- Textured<br>- Plain<br>- Dynamic<br>- Open form<br>- Painterly	- Linear<br>- Closed form<br>- Formless<br>- Chaotic<br>- Brush strokes<br>- Architectural<br>- Industrial<br>- Abstract	- Decorative<br>- Pattern<br>- Floral<br>- Text/calligraphy

Composition			Subject Matter		
- Open composition<br>- Closed composition<br>- Symmetrical<br>- Tendency toward symmetry<br>- Asymmetrical<br>- Centered alignment of the subject matter	- Horizontal frame<br>- Vertical frame<br>- Square frame<br>- Multiple frames<br>- Emphasis on a single subject matter	- Busy and crowded compositions<br>- Empty and quiet composition	- Human body<br>- Female body<br>- Male body<br>- Human torso<br>- Male torso<br>- Female torso<br>- Portraits<br>- Hidden human faces	- Still life<br>- Animals<br>- Nature/landscape<br>- Architecture<br>- Interior space<br>- Industrial space<br>- Urban spaces	- Domestic space<br>- Shadows, reflections<br>- Everyday objects<br>- Graffiti<br>- Dolls/toys<br>- Cars
