# Supplementary material for: How deep is your art: An experimental study on the limits of artistic understanding in a single-task, single-modality neural network
Source: PLoS One. 2024 Nov 6;19(11):e0305943. doi: 10.1371/journal.pone.0305943 (PMC11540182; doi:10.1371/journal.pone.0305943)
Supplement: S2 Table — (PDF) [file pone.0305943.s002.pdf]

Table 2. Galleries' EXPs

Gallery	EXPs			
	**Medium, Color**	**Shape, Form, Texture**	**Composition**	**Subject Matter**
30 Years of Women	- Black and white photography<br>- Color photography<br>- Mix media, using photography<br>- High saturation<br>- Medium saturation<br>- Low saturation<br>- Cool colors<br>- Warm colors<br>- Neutral colors<br>- Dark value<br>- Light value<br>- Mid-tones<br>- High contrast<br>- Low contrast<br>- Medium contrast<br>- Monochromatic<br>- Chromatic<br>- Colorful	- Figurative<br>- Organic<br>- Geometric textured<br>- Plain<br>- Dynamic<br>- Open form<br>- Painterly linear<br>- Closed form<br>- Formless chaotic brush strokes<br>- Architectural<br>- Industrial<br>- Abstract decorative pattern<br>- Floral<br>- Text/calligraphy	- Pen composition<br>- Closed composition<br>- Symmetrical<br>- Tendency toward symmetry<br>- Asymmetrical<br>- Centered alignment of the subject matter<br>- Horizontal frame<br>- Vertical frame<br>- Square frame<br>- Emphasis on a single subject matter<br>- Busy and crowded compositions<br>- Empty and quiet composition	- Human body<br>- Female body<br>- Male body<br>- Human torso<br>- Female torso<br>- Male torso<br>- Portraits<br>- Hidden human faces<br>- Still life<br>- Animals<br>- Nature/landscape<br>- Architecture<br>- Interior space<br>- Industrial space<br>- Domestic space<br>- Shadows, reflections<br>- Everyday objects<br>- Graffiti
Boarding House	-Black and white photography<br>- Low saturation<br>- Neutral colors<br>- Dark value<br>- Light value<br>- Mid-tones<br>- Medium contrast<br>- Monochromatic	- Organic<br>- Figurative<br>- Textured<br>- Open form<br>- Closed form<br>- Linear<br>- Repetitions of a shape or an element<br>- Pattern	- Square frame<br>- Busy/crowded compositions<br>- Open composition<br>- Closed composition<br>- Tendency toward symmetry<br>- Asymmetrical<br>- Centered alignment of the subject matter<br>- Empty and quiet composition	- Human body<br>- Still life<br>- Animals<br>- Nature/landscape<br>- Abandoned urban space<br>- Graffiti<br>- Everyday objects<br>- Dolls/toys
Bonsai	- Black and white photography<br>- Monochromatic<br>- Warm tone<br>- Neutral colors<br>- Dark value<br>- Light value<br>- Mid-tones<br>- High contrast<br>- Low contrast<br>- Medium contrast	- Organic shapes<br>- Natural<br>- Textured<br>- Plain<br>- Open forms<br>- Painterly<br>- Formless<br>- Abstract	- Close composition<br>- Asymmetrical composition<br>- Tendency toward symmetrical composition<br>- Vertical composition<br>- Horizontal composition<br>- Simple and quiet<br>- Centered alignment of the subject matter<br>- Emphasis on a single subject matter	- Landscape/nature<br>- Animals/ owls<br>- Shadows/reflections

Bullets	- Colored photography<br>- Limited colors<br>- Medium saturation<br>- Low saturation<br>- Warm colors<br>- Neutral colors<br>- Dark value<br>- Light value<br>- Mid-tone<br>- Medium contrast<br>- Chromatic	- Figurative<br>- Organic<br>- Textured<br>- Closed forms<br>- Decorative pattern<br>-Text/calligraphy	- Horizontal compositions<br>- Vertical compositions<br>- Multi-frames<br>- Closed composition<br>- Asymmetrical<br>- Tendency toward symmetry<br>- Centered alignment<br>- Busy and crowded composition	- Female body<br>- Arabic calligraphy /text<br>- Interior space
Close	- Color photography<br>- High saturation<br>- Medium saturation<br>- Cool colors<br>- Warm colors<br>- Dark value<br>- Light value<br>- Mid-tones<br>- High contrast<br>- Medium contrast<br>- Chromatic<br>- Colorful	- Figurative<br>- Organic<br>- Geometric<br>- Open form<br>- Closed form<br>- Architectural<br>- Decorative<br>- Pattern<br>- Floral	- Busy and crowded<br>- Asymmetrical<br>- Square frames<br>- Open composition<br>- Closed composition<br>- Tendency toward symmetry<br>- Centered alignment of the subject matter<br>- Emphasis on a single subject	- Human body<br>- Female body<br>- Male body<br>- Human torso<br>- Male torso<br>- Female torso<br>- Portraits<br>- Hidden human faces<br>- Nature/landscape<br>- Architecture<br>- Interior space<br>- Domestic space<br>- Shadows, reflections
Converging Territories	- Color photography (coupler printed)<br>- Medium saturation<br>- Low saturation<br>- Cool colors<br>- Warm colors<br>- Neutral colors<br>- Dark value<br>- Light value<br>- Mid-tones<br>- Low contrast<br>- Medium contrast<br>- Chromatic	- Figurative<br>- Textured surfaces<br>- Repetition of Arabic calligraphic elements<br>- Open forms<br>- Closed forms<br>- Linear<br>- Decorative pattern	- Vertical frames<br>- Horizontal frames<br>- Multiple frames<br>- Symmetrical<br>- Asymmetrical<br>- Centered alignment of the subject matter<br>- Emphasis on a single subject matter	- Female body<br>- Portraits<br>- Hidden human faces<br>- Still life<br>- Arabic calligraphy<br>- Female veils
Eat Flowers	- Color photography<br>- High saturation<br>- Medium saturation<br>- Low saturation<br>- Cool colors<br>- Warm colors<br>- Neutral colors<br>- Dark value<br>- Light value<br>- Mid-tones<br>- High contrast<br>- Low contrast<br>- Medium contrast<br>- Monochromatic<br>- Chromatic<br>- Colorful	- Organic and natural<br>- Floral<br>- Figurative<br>- Text/ letters<br>- Plain<br>- Textured<br>- Abstract<br>- Decorative<br>- Formless<br>- Open form<br>- Painterly<br>- Linear	- Open composition<br>- Closed composition<br>- Tendency toward symmetry<br>- Asymmetrical<br>- Centered alignment of the subject matter<br>- Horizontal frame<br>- Vertical frame<br>- Emphasis on a single subject matter<br>- Busy and crowded compositions<br>- Empty and quiet composition	- Human body<br>- Female body<br>- Male body<br>- Human torso<br>- Portraits<br>- Interior spaces<br>- Hidden human faces<br>- Still life<br>- Animals<br>- Nature/landscape<br>- Shadows, reflections<br>- Everyday objects<br>- Dolls/toys<br>- Cars<br>- Text/calligraphy<br>- Fruits

Epilogue	- Color photography<br>- Black and White photography<br>- High saturation<br>- Medium saturation<br>- Low saturation<br>- Cool colors<br>- Warm colors<br>- Neutral colors<br>- Dark Value<br>- Light value<br>- Mid-tones<br>- High contrast<br>- Low contrast<br>- Medium contrast<br>- Monochromatic<br>- Chromatic<br>- Colorful	- Figurative<br>- Organic<br>- Geometric<br>- Textured<br>- Plain<br>- Dynamic<br>- Open form<br>- Linear<br>- Closed form<br>- Formless<br>- Chaotic<br>- Architectural<br>- Industrial<br>- Abstract<br>- Pattern<br>- Floral	- Vertical<br>- Horizontal<br>- Squares<br>- Asymmetrical<br>- Near symmetrical<br>- Crowded and busy<br>- Empty and simple<br>- Open composition<br>- Closed composition<br>- Centered alignment of the subject matter<br>- Multiple frames<br>- Emphasis on a single subject matter	- Human body<br>- Portraits<br>- Still life<br>- Nature/landscape<br>- Architecture<br>- Interior space<br>- Industrial space<br>- Urban spaces<br>- Domestic space<br>- Shadows, reflections<br>- Everyday/objects<br>- Dolls/toys
Evidence	- Color photography<br>- High Saturation<br>- Medium saturation<br>- Cool colors<br>- Warm colors<br>- Dark Value<br>- Light value<br>- Mid-tones<br>- High Contrast<br>- Medium contrast<br>- Chromatic<br>- Colorful<br>- Dramatic<br>- Monochromatic	- Figurative<br>- Organic<br>- Geometric<br>- Open form<br>- Closed form<br>- Architectural<br>- Pattern<br>- Floral<br>- Dramatic lights	- Busy and crowded<br>- Empty and quiet<br>- Asymmetrical<br>- Square frames<br>- Open composition<br>- Closed composition<br>- Tendency toward symmetry<br>- Centered alignment of the subject matter<br>- Emphasis on a single subject	- Human body<br>- Female body<br>- Male body<br>- Human torso<br>- Male torso<br>- Female torso<br>- Portraits<br>- Hidden human faces<br>- Nature/landscape<br>- Architecture<br>- Interior space<br>- Domestic space<br>- Shadows, reflections<br>- Dramatic light
Familiar Landscapes	- Color photography<br>- Medium saturation<br>- High saturation<br>- Cool colors<br>- Warm colors<br>- Dark Value<br>- Light value<br>- Mid-tones<br>- High Contrast<br>- Medium contrast<br>- Chromatic<br>- Colorful	- Organic<br>- Textured<br>- Open form<br>- Painterly<br>- Formless<br>- Floral	- Horizontal<br>- Vertical<br>- Asymmetrical<br>- Fairly busy and crowded	- Nature/landscape<br>- Light<br>- Spring

Heat + High Fashion	- Black and white photography<br>- Monochromatic<br>- Dark Value<br>- Light value<br>- Midtones<br>- High Contrast	- Figurative<br>- Organic<br>- Plain<br>- Open forms<br>- Painterly	- Closed compositions<br>- Open composition<br>- Tendency toward symmetry<br>- A-symmetrical<br>- Centered<br>- Alignment of the subject matter<br>- Horizontal frames<br>- Vertical frames<br>- Emphasis on a single subject matter<br>- Empty and quiet composition<br>- Busy and crowded compositions	- Female body<br>- Female torso<br>- Portraits<br>- Hidden human faces<br>- Fashion<br>- Interior space<br>- Shadows, reflections
Hivernacle	- Mixed media (photography and paint)<br>- Black, white, grays + gold<br>- Medium saturation<br>- Low saturation<br>- Warm colors<br>- Neutral colors<br>- Dark Value<br>- Light value<br>- Mid-tones<br>- High Contrast<br>- Medium contrast<br>- Monochromatic	- Figurative<br>- Organic<br>- Plain<br>- Open forms<br>- Closed forms<br>- Brush strokes	- Closed composition<br>- Tendency toward symmetry<br>- Asymmetrical<br>- Centered alignment of the subject matter<br>- Horizontal frame<br>- Vertical frame<br>- Emphasis on a single subject matter<br>- Empty and quiet composition	- Female body<br>- Female torso<br>- Hidden human faces
Kawa = Flow	- Black and white photography<br>- Color Photography<br>- Monochromatic<br>- Photography<br>- Cool colors<br>- Warm colors<br>- Dark Value<br>- Light value<br>- Mid-tones<br>- Low saturated colors<br>- Neutral colors<br>- High Contrast<br>- Low contrast<br>- Medium contrast	- Figurative shapes<br>- Organic shapes<br>- Textured<br>- Plain<br>- Open forms<br>- Painterly<br>- Formless<br>- Abstract<br>- Floral	- Open composition<br>- Close composition<br>- Asymmetrical composition<br>- Symmetrical composition<br>- Vertical composition<br>- Horizontal composition<br>- Square composition<br>- Busy and crowded composition<br>- Simple and quiet<br>- Centered alignment of the subject matter<br>- Emphasis on a single subject matter	- Landscape/nature<br>- Human body<br>- Female body<br>- hidden human faces<br>- Still life<br>- Animals<br>- Shadows/reflections<br>- Human and nature relationship
Little Deaths	- Color photography<br>- High saturated<br>- Colors<br>- Low saturated colors<br>- Medium to high contrast<br>- Medium saturation<br>- Cool colors<br>- Warm colors<br>- Neutral colors<br>- Chromatic<br>- Colorful	- Natural<br>- Organic<br>- Textured<br>- Open form<br>- Painterly<br>- Formless<br>- Chaotic<br>- Floral	- Asymmetrical horizontal<br>- Vertical<br>- Closed compositions<br>- Centered alignment of the subject matter<br>- Emphasis on a single subject matter<br>- Busy and crowded compositions<br>- Domestic space	- Landscape/ nature<br>- Animal<br>- Human body<br>- Female body<br>- Male body<br>- Human torso

Mukono	- Black and white photography<br>- Monochromatic<br>- Dark Value<br>- Light value<br>- Midtones<br>- High Contrast<br>- Medium contrast<br>- Low contrast<br>- Low saturation<br>- Neutral colors	- Figurative<br>- Organic<br>- Plain<br>- Open forms<br>- Closed forms	- Closed composition<br>- Tendency toward symmetry<br>- Asymmetrical<br>- Centered alignment of the subject matter<br>- Horizontal frame<br>- Vertical frame<br>- Emphasis on a single subject matter<br>- Empty and quiet composition	- Human torso/male torso<br>- Female torso<br>- Portraits<br>- Hidden human faces<br>- Nature/landscape<br>- Human body<br>- Everyday objects<br>- Still life<br>- Animals
My Mother's Clothes	- Color photography<br>- High Saturation<br>- Medium saturation<br>- Low saturation<br>- Cool colors<br>- Warm colors<br>- Neutral colors<br>- Dark Value<br>- Light value<br>- Midtones<br>- High Contrast<br>- Low contrast<br>- Medium contrast<br>- Chromatic	- Organic<br>- Geometric<br>- Textured<br>- Plain<br>- Open form<br>- Linear<br>- Closed form<br>- Decorative<br>- Pattern<br>- Floral<br>- Text	- Closed composition<br>- Tendency toward symmetry<br>- Asymmetrical<br>- Centered alignment of the subject matter<br>- Square frames<br>- Emphasis on a single subject matter<br>- Busy and crowded compositions<br>- Empty and quiet composition	- Female clothes<br>- Still life<br>- Everyday objects<br>- Domestic space
Native	- Color photography<br>- High Saturation<br>- Medium saturation<br>- Cool colors<br>- Warm colors<br>- Dark Value<br>- Light value<br>- Mid-tones<br>- High Contrast<br>- Medium contrast<br>- Chromatic<br>- Colorful	- Figurative<br>- Organic<br>- Open form<br>- Closed form<br>- Architectural<br>- Decorative<br>- Pattern<br>- Floral<br>- Textured<br>- Formless<br>- Abstract<br>- Pattern	- Busy and crowded<br>- Empty and quiet<br>- Asymmetrical<br>- Square frames<br>- Open composition<br>- Closed composition<br>- Tendency toward symmetry<br>- Centered alignment of the subject matter<br>- Emphasis on a single subject	- Nature/landscape<br>- Human body<br>- Female body<br>- Male body<br>- Human torso<br>- Male torso<br>- Female torso<br>- Portraits<br>- Hidden human faces<br>- Interior space<br>- Domestic space<br>- Shadows, reflections<br>- Human in nature
New York, Paris, and Rome	- Black and white photography<br>- Medium to high contrast<br>- Monochromatic<br>- Neutral colors<br>- Dark value<br>- Light value<br>- Mid-tones	- Figurative<br>- Urban<br>- Architectural<br>- Organic<br>- Natural and organic<br>- Open forms<br>- Close forms<br>- Dynamic<br>- Formless<br>- Industrial text	- Open composition<br>- Closed composition<br>- Tendency toward symmetry<br>- Asymmetrical<br>- Centered alignment of the subject matter<br>- Horizontal frame<br>- Vertical frame<br>- Square frame<br>- Busy and crowded compositions	- Human body<br>- Female body<br>- Male body<br>- Human torso<br>- Portraits<br>- Hidden human faces<br>- Animals<br>- Nature/landscape<br>- Architecture<br>- Industrial space<br>- Urban spaces<br>- Shadows, reflections<br>- Cars

Painted Nudes	- Mixed Media (combination of photography and painting)<br>- High Saturation<br>- Medium saturation<br>- Low saturation<br>- Cool colors<br>- Warm colors<br>- Neutral colors<br>- Dark Value<br>- Light value<br>- Mid-tones<br>- High contrast<br>- Medium contrast<br>- Chromatic<br>- Color full	- Figurative<br>- Textured<br>- Dynamic brush strokes<br>- Open form<br>- Painterly<br>- Brush strokes<br>- Abstract<br>- Formless<br>- Chaotic	- Open composition<br>- Closed composition<br>- Asymmetrical<br>- Horizontal frame<br>- Vertical frame<br>- Busy and crowded compositions<br>- Centered alignment of the subject matter	- Female body<br>- Female torso
Paradise Lost	- Color photography<br>- High Saturation<br>- Medium saturation<br>- Cool colors<br>- Warm colors<br>- Dark Value<br>- Light value<br>- Mid-tones<br>- High Contrast<br>- Medium contrast<br>- Chromatic<br>- Colorful	- Figurative<br>- Organic<br>- Geometric<br>- Open form<br>- Closed form<br>- Architectural<br>- Decorative<br>- Pattern<br>- Floral	- Busy and crowded<br>- Asymmetrical<br>- Square frames<br>- Open composition<br>- Closed composition<br>- Tendency toward symmetry<br>- Centered alignment of the subject matter<br>- Emphasis on a single subject	- Human body<br>- Female body<br>- Male body<br>- Human torso<br>- Male torso<br>- Female torso<br>- Portraits<br>- Hidden human faces<br>- Nature/landscape<br>- Architecture<br>- Interior space<br>- Domestic space<br>- Shadows, reflections
Persephone	- Mixed media (photography + paint)<br>- High Saturation<br>- Medium saturation<br>- *Ed colors<br>- Cool colors<br>- Warm colors<br>- Neutral color<br>- Dark Value<br>- Light value<br>- Mid-tones<br>- Medium contrast<br>- Chromatic<br>- Colorful	- Organic<br>- Open form<br>- Textured<br>- Painterly<br>- Formless<br>- Chaotic<br>- Brush strokes<br>- Abstract<br>- Floral<br>- Abstract	- Open composition<br>- Closed composition<br>- Asymmetrical<br>- Horizontal frame<br>- Vertical frame<br>- Busy and crowded compositions	- Landscape/nature

Private	- Color photography<br>- Brownish dominant color<br>- Medium saturation<br>- Warm colors<br>- Neutral colors<br>- Dark Value<br>- Light value<br>- Mid-tones<br>- Medium contrast<br>- Monochromatic<br>- Chromatic	- Figurative<br>- Organic<br>- Textured<br>- Plain<br>- Open form<br>- Closed form<br>- Formless<br>- Abstract<br>- Pattern<br>- Text	- Square frames<br>- Open composition<br>- Closed composition<br>- Symmetrical<br>- Tendency toward symmetry<br>- Asymmetrical<br>- Centered alignment of the subject matter<br>- Emphasis on a single subject matter<br>- Busy and crowded<br>- Compositions<br>- Empty and quiet composition	- Human body<br>- Female body<br>- Male body<br>- Human torso<br>- Male torso<br>- Female torso<br>- Hidden human faces<br>- Nature/landscape<br>- Interior space<br>- Domestic space<br>- Shadows, reflections
Scene	- Black and white photography<br>- Monochromatic<br>- Dark Value<br>- Light value<br>- Midtones<br>- High Contrast<br>- Medium contrast	- Figurative<br>- Textured<br>- Plain<br>- Open form<br>- Closed-form<br>- Pattern	- Closed composition<br>- Tendency toward symmetry<br>- Asymmetrical<br>- Centered alignment of the subject matter<br>- Square frame<br>- Emphasis on a single subject matter	- Human body<br>- Female body<br>- Male body<br>- Human torso<br>- Male torso<br>- Female torso<br>- Interior space<br>- Shadows, reflections<br>- Artists
Sweet 16	- Color photography<br>- High Saturation<br>- Medium saturation<br>- Cool colors<br>- Warm colors<br>- Neutral colors<br>- Chromatic<br>- Colorful<br>- Dark Value<br>- Light value<br>- Mid-tones<br>- High Contrast	- Figurative<br>- Texture/pattern on the background<br>- Floral<br>- Decorative<br>- Organic	- Centered figures<br>- Vertical<br>- Tendency toward symmetry<br>- Closed compositions<br>- Emphasis on a single subject matter	- Female torso<br>- Portraits<br>- Interior space
The Fall of Spring Hill	- Color photography<br>- High Saturation<br>- Medium saturation<br>- Cool colors<br>- Warm colors<br>- Neutral colors<br>- Dark Value<br>- Light value<br>- Mid-tones<br>- High Contrast<br>- Medium contrast<br>- Chromatic	- Figurative<br>- Organic<br>- Figurative<br>- Organic<br>- Textured<br>- Plain<br>- Dynamic<br>- Closed form<br>- Architectural	- Open composition<br>- Closed composition<br>- Asymmetrical<br>- Horizontal frame<br>- Emphasis on a single subject matter<br>- Busy and crowded compositions	- Human body<br>- Female body<br>- Children<br>- Human torso<br>- Nature/landscape<br>- Interior spaces<br>- Domestic spaces<br>- Shadow/reflections<br>- Everyday objects<br>- Still-life

The Fallen Fawn	- Color photography<br>- Medium saturated colors<br>- High saturated colors<br>- Medium contrast	- Figurative<br>- Everyday objects<br>- Interior spaces<br>- Organic<br>- Natural<br>- Open form<br>- Painterly<br>- Closed form<br>- Chaotic<br>- Decorative<br>- Pattern<br>- Floral	- Horizontal<br>- Asymmetrical<br>- Busy and crowded<br>- Open composition<br>- Emphasis on a single subject matter	- Human body<br>- Female body<br>- Male body<br>- Human torso<br>- Male torso<br>- Female torso<br>- Portraits<br>- Still life<br>- Animals<br>- Nature/landscape<br>- Interior space<br>- Domestic space<br>- Shadows, reflections<br>- Everyday objects<br>- Dolls/toys<br>- Cars
The Garden	- Color photography<br>- Diverse colors<br>- Cool colors<br>- Warm colors<br>- Neutral colors<br>- Dark Value<br>- Light value<br>- Mid-tones<br>- High Contrast<br>- Medium contrast<br>- Chromatic<br>- Colorful	- Figurative organic<br>- Textured<br>- Plain<br>- Dynamic<br>- Open form<br>- Painterly<br>- Closed form<br>- Formless<br>- Chaotic<br>- Abstract<br>- Decorative<br>- Pattern<br>- Floral	- Closed composition<br>- Symmetrical<br>- Tendency toward symmetry<br>- Asymmetrical<br>- Centered alignment of the subject matter<br>- Horizontal frame<br>- Vertical frame<br>- Emphasis on a single subject matter<br>- Busy and crowded compositions<br>- Empty and quiet composition	- Human body<br>- Female body<br>- Female torso<br>- Portraits<br>- Hidden human faces<br>- Nature/landscape<br>- Shadows, reflections
The Unknown	- Mixed media photography<br>- Diverse colors<br>- Cool colors<br>- Warm colors<br>- Neutral colors<br>- Dark Value<br>- Light value<br>- Mid-tones<br>- High Contrast<br>- Medium contrast<br>- Chromatic<br>- Colorful	- Figurative<br>- Organic<br>- Textured<br>- Plain<br>- Open form<br>- Painterly<br>- Closed form<br>- Brush stroke	- Closed composition<br>- Symmetrical<br>- Tendency toward symmetry<br>- Asymmetrical<br>- Centered alignment of the subject matter<br>- Horizontal frame<br>- Vertical frame<br>- Emphasis on a single subject matter<br>- Busy and crowded compositions<br>- Empty and quiet composition	- Human body<br>- Female body<br>- Female torso<br>- Portraits<br>- Hidden human faces

Trigger	- Color photography<br>- Medium saturation<br>- Low saturation<br>- Neutral colors<br>- Cool colors<br>- Dark value<br>- Light value<br>- Mid-tones<br>- High Contrast<br>- Low contrast<br>- Medium contrast<br>- Chromatic	- Plain<br>- Organic<br>- Geometric<br>- Closed form	- Closed composition<br>- Tendency toward symmetry<br>- Asymmetrical<br>- Centered alignment of the subject matter<br>- Horizontal frame<br>- Vertical frame<br>- Emphasis on a single subject matter<br>- Empty and quiet composition	- Interior space<br>- Domestic space<br>- Still life<br>- Animals
