# Supplementary material for: How deep is your art: An experimental study on the limits of artistic understanding in a single-task, single-modality neural network
Source: PLoS One. 2024 Nov 6;19(11):e0305943. doi: 10.1371/journal.pone.0305943 (PMC11540182; doi:10.1371/journal.pone.0305943)
Supplement: S3 Table — (PDF) [file pone.0305943.s003.pdf]

Table 3. Galleries' NEXPs

Gallery	NEXPs		
	**Context**	**Intention**	**Meaning**
30 Years of Women	- Women art 20-21 centuries photographers<br>- Global/cross-cultural<br>- Contemporary<br>- Feminism	- Modern and contemporary women art<br>- Female gaze, female identity	- Female body<br>- Female gaze<br>- Identity<br>- female perspective and gaze<br>- Female body (not all)<br>- Global feminism<br>- Sensuality<br>- Female fashion<br>- Ethnicity and cultural identity<br>- Realism and indexicality<br>- Intersection of feminism, gender, and sexuality
Boarding House	- Contemporary, American and South African<br>- Jewish culture<br>- Contemporary surrealism	- Surrealism art, or art that relates to human psyche<br>- Indexicality	- A space between reality and fantasy [1]<br>- Human existence in relation to place and environment [1]<br>- Outmoded objects, abandoned places, and things, elements that eco-surrealism in art and film
Bonsai	- Japanese modern and contemporary art<br>- Zen philosophy [2]<br>- Romanticism<br>- Traditions of landscape art	- Contemporary Japan<br>- Japanese philosophy of Zen<br>- Painterly quality in photography, meaning a photography that is poetic and subjective vs. Realistic<br>- Being one with nature, monism of human and nature, mind, and body	- Zen philosophy<br>- The relationship between human and nature, human as a part of the nature or in a monistic relation with nature<br>- Female body as a metaphor of a landscape<br>- Being one with nature, the monism of human and nature, mind and body<br>- A dream-like quality<br>- *Strong emotions and subjective expression
Bullets	- Orientalism<br>- Globalization<br>- Contemporary photography<br>- Middle eastern art<br>- Imperialism/ colonialism	- Middle eastern identity in relation to western orientalism and imperialism [3]<br>- Identity politics in visual art and culture	- Orientalist representation of the artist's cultural identity<br>- Identity politics
Close	- German-Brazilian contemporary photographer<br>- Traditional nude imagery rooted in Western classical and academic art	- Aesthetics and beauty of the human body, inspired by the Western classical art historical discourse of representation of a nude figure<br>- Intimate connection, intimate space with human figures	- Intimate depiction of the models [4]<br>- A natural state of the human body, and reflecting being comfortable in one own's skin [4]<br>- Sensual quality<br>- Formal beauty, often stereotypical, traditional and classical idea of beauty<br>- Human body integrated or in a monistic relationship with nature
Converging Territories	- Arab cultures<br>- Islamic culture<br>- Arab-American<br>- Modern and contemporary<br>- Orientalism<br>- Imperialism<br>- Globalization	- Contemporary Arab American art [3]<br>- Art that challenges Western Orientalism [3]<br>- Immigrant's identity in American [3]	- Challenging the stereotypes about Arab and Muslim women [3]<br>- Challenging social, and cultural hierarchies [3]<br>- Arab women Identity [3]<br>- Islamic culture [3]

Eat Flowers	- Contemporary British Art<br>- Contemporary American art<br>- Conceptual art	- Contemporary European and American art<br>- Representation of nature and the human relationship with nature<br>- "I want my photographs to be a jolt, they explore the magic in the world while having one foot very much placed in reality." [5]<br>- Conceptual art	- "Natural environment and familial relationships" [5]<br>- Influence by the natural environment of her hometown [5]<br>- Poetic<br>- Dreamlike<br>- Memory<br>- Time
Epilogue	- Modern and contemporary photography<br>- Cross-cultural<br>- Realism<br>- Surrealism<br>- Pop art and culture	- Contemporary<br>- Realism<br>- Surrealism art<br>- Pop art and culture	- Documenting aftermath of an event<br>- Realism and indexicality<br>- Epilogue (the title of the show) meaning "the moment after." According to the gallery, the moment after refers to documenting a moment as an event. Recording the event is in fact a record of the aftermath of the event [6].<br>- Outmoded objects<br>- A world between fantasy and reality<br>- Memory and time
Evidence	- German-Brazilian contemporary photographer<br>- Traditional nude imagery rooted in Western classical and academic art	- Aesthetics and beauty of the human body, inspired by the Western classical art historical discourse of representation of nude figure<br>- Intimate connection<br>- Intimate space with human figures	- Intimacy and intimate depiction of the models [4]<br>- A natural state of the human body, and reflecting being comfortable in one own's skin [4]<br>- Sensual quality<br>- Formal Beauty, often stereotypical, traditional and classical idea of beauty<br>- Human body integrated or in a monistic relationship with nature
Familiar Landscapes	- Contemporary American photography<br>- Landscape art<br>- Realism/romanticism, contemporary	- Landscape art<br>- Her hometown and its nature	- Her hometown and the chronology of its inhabitants [7]<br>- The nature and place of community [7]<br>- Passing of time [7]<br>- A sense of romanticism through dramatic light in nature
Heat + High Fashion	- Modern contemporary<br>- New York, "bohemian."	- Engaging female fashion through photography<br>- Realism yet a sense of ambiguity through blurred images and painterly qualities of the medium of photography (in that sense it can place itself against modernist photography and its notion of medium specificity)	- Photographs of dancer Isadora Duncan<br>- Fashion photography that reflects the time and historical context<br>- Female identity through fashion and clothing<br>- Realism yet a sense of ambiguity through blurred images and painterly qualities of the medium of photography
Hivernacle	- Contemporary Spanish art<br>- Fine arts photography inspired by fashion photography<br>- Female identity in the contemporary art<br>- Surrealism	- Contemporary<br>- Issue of identity, and identity<br>- Fine art photography inspired by fashion<br>- Dream like portraits<br>- Ambiguous portraits<br>- Artistic tradition of portraiture	- Ambiguous identities (From the title)<br>- Human psyche<br>- Influenced by surrealism and psychoanalysis<br>- Relation between dream and reality<br>- Female identity<br>- Ambiguity<br>- Portraiture

Kawa = Flow	- Japanese modern and contemporary art<br>- Zen philosophy<br>- Romanticism<br>- Traditions of landscape art	- Contemporary Japan [2]<br>- Japanese philosophy of Zen [2]<br>- Painterly quality in photography, meaning photography that is poetic and subjective vs. realistic<br>- Being one with nature, the monism of human and nature, mind and body	- Zen philosophy<br>- The relationship between human and nature, human as a part of the nature or in a monistic relation with nature<br>- Female body as a metaphor of a landscape<br>- Being one with nature, the monism of human and nature, mind and body<br>- A dream-like quality<br>- Strong emotions and subjective expression
Little Deaths	- Contemporary American photography<br>- Landscape art<br>- Realism/romanticism, contemporary	- Landscape art<br>- Her hometown and its nature	- Her hometown and the chronology of its inhabitants [7]<br>- The nature and place of community [7]<br>- Passing of time [7]<br>- A sense of romanticism through dramatic light in nature
Mukono	- Contemporary photography<br>- Cultural studies	- Realism and documentary photography	- Documenting people around the world<br>- Race, ethnicity, culture-racial and cultural identity
My Mother's Clothes	- Contemporary photography and conceptual art (using ready-made objects as works of art)	- Conceptual photography inspired by conceptual art and the use of ready-made/ordinary objects and blurring the boundary between art and life<br>-Photographing her mothers' clothes and personal idem as a form of portraits or chronology of her mother's life<br>- Using art and photography to cope with the loss of her mother and her mother's suffering from Alzheimer	- Her mother's clothes and personal items as her mother's portrait/body= clothes as a metonymy of the person<br>- Remembering the past, memories of her mother, perhaps a sense of nostalgia<br>- Coping with Truma of loss of her mother and her suffering from Alzheimer<br>- Gender expression/identity<br>- Social class in America
Native	- German-Brazilian contemporary photographer<br>- Photography influenced by the tradition of academic and representational painting	- "a personal journey. Metaphorically, I was thinking of a bird that flies back into the forest, searching for its childhood." [8]<br>- Aesthetics and beauty of the human body, inspired by the Western classical art historical discourse of representation of nude figure [8]<br>- Intimate connection, intimate space with human figures [8]	- "a personal journey. Metaphorically, I was thinking of a bird that flies back into the forest, search for its childhood."<br>- "Abstract wishes and dreams"<br>- "Her native country" to re-interpret her past<br>- Intimacy and se intimate depiction of the models<br>- A natural state of the human body, and reflecting being comfortable in one own's skin<br>- Sensual quality<br>- Formal beauty, often stereotypical, traditional and classical idea of beauty<br>- Human body integrated or in a monistic relationship with nature
New York, Paris, and Rome	- Modern and contemporary art<br>- American Art<br>- Realism<br>- Documentary photography	- Realism<br>- Documentary and indexicality in photography<br>- Europe and North American art in the mid-late 20th century<br>- Capturing a moment in time through photography	- Documentary photography<br>- Urban everyday life<br>- Realism<br>- Indexicality<br>- Human relationships with their environments<br>- Artistic and cultural scenes in New York, Paris, and Rome

Painted Nudes	- Contemporary art<br>- American art	- Contemporary American mixed media art<br>- Experimental approach to the medium of photography<br>- Formal beauty	- Searching for beauty in simple and everyday things [9]<br>- Eroticism
Paradise Lost	- German-Brazilian contemporary photographer	- Aesthetics and beauty of the human body, inspired by the Western classical art historical discourse of representation of nude figure<br>- Intimate connection, intimate space with human figures	- Intimate depiction of the models [4]<br>- A natural state of the human body, and reflecting being comfortable in one own's skin [4]<br>- Sensual quality<br>- Formal beauty, often stereotypical, traditional and classical idea of beauty<br>- Human body integrated or in a monistic relationship with nature
Persephone	- Contemporary American nature	- Contemporary<br>- Contemporary American towns and communities<br>- Contemporary landscape mixed media art<br>- Representation of her hometown, a sense of community and the story of people who have lived there	- "her hometown and the chronology of its inhabitant" [7]<br>- An imaginative depiction of landscape vs a simple realism<br>- The brush strokes can show the passage of time or artist's subjective feeling to her environment
Private	- German-Brazilian contemporary photographer	- Aesthetics and beauty of the human body, inspired by the Western classical art historical discourse of representation of nude figure<br>- Intimate connection, intimate space with human figures	- Intimate depiction of the models [4]<br>- A natural state of the human body, and reflecting being comfortable in one own's skin [4]<br>- Sensual quality<br>- Formal beauty, often stereotypical, traditional and classical idea of beauty<br>- Human body integrated or in a monistic relationship with nature
Scene	- 1960s underground/avant-garde artists' scenes in the United States	- Realistic photographs of Avant-garde artists in New York during the 1960s<br>- Documentary photography	- Photography and realism<br>- Indexicality<br>- Representation of avant-garde artists in NYC during the c1960s<br>- Human emotion and psychological expression
Sweet 16	- Contemporary American photography<br>- Tradition of portrait art<br>- Realism	- Contemporary, American<br>- Realism/representational art<br>- Tradition of portraiture painting, photography<br>- Realism	- Engaging the historical tradition of portraiture<br>- The identity of the model the way they want to be perceived and the way they are perceived by the camera [10]<br>- Her hometown and the chronology of its inhabitants. [10]<br>- The nature and place of community [10]<br>- Passing of time
The Fall of Spring Hill	- Contemporary American photography<br>- Life and culture in the suburbs/small town in America<br>- Representational art	- Contemporary photography<br>- Representation of life in suburbs/small towns in America<br>- "Cinematic Scenarios an incident from a summer church camp in which a child injures himself by falling from a dilapidated wooden play structure and the mothers' fierce reaction to deconstruct it in retribution." [11]	- Creating cinematic scenarios<br>- Representation of memory through photography<br>- Life and culture in the suburbs/small town in America<br>- Domestic life<br>- Performativity in photography

The Fallen Fawn	- Contemporary American photography<br>- Life and culture in the suburbs/small town in America<br>- Representational art<br>- Contemporary film and cinema<br>- Narrative art	- Contemporary photography [11]<br>- Representation of life in suburbs/small towns in America [11]<br>- "Cinematic Scenarios an incident from a summer church camp in which a child injures himself by falling from a dilapidated wooden play structure and the mothers' fierce reaction to deconstruct it in retribution." [11]	- Creating cinematic scenarios [11]<br>- Representation of memory through photography [11]<br>- Life and culture in the suburbs/small town in America [11]<br>- Domestic life [11]<br>- Performativity in photography [11]
The Garden	- Contemporary Spanish art and photography<br>- Subjective images through photography vs. Realism	- Subjective expression and painterly qualities through photography rather using photography as a medium of realism	- Nature and female body<br>- Mysterious and ambiguous identities and environments<br>- Psychological expression through photography vs. Realism<br>- Metaphor of female body as landscape<br>- A sense of timelessness
The Unknown	- Contemporary Spanish art<br>- Fashion photography	- Photography and art inspired by fashion photography Contemporary<br>- Issue of identity, ambiguity	- Ambiguous identities (From the title)<br>- Human psyche<br>- Influenced by surrealism and psychoanalysis<br>- Relation between dream and reality<br>- Female identity<br>- Ambiguity<br>- Portraiture
Trigger	- Contemporary photography<br>- Conceptual photography	- Conceptual photography<br>- Photographing everyday objects and domestic space (her hometown)<br>- Capturing time passing through photography	- Artist' hometown and the lives of people who has lived there -CITE-"Passing of time"<br>- Her personal experiences<br>- Collision of past and present<br>- Domestic space and meaningful—perhaps personal —everyday lives objects<br>- Time, temporality

References

1. Roger Ballen- Boarding House, October 29 - December 24, 2010;. Available from: `https://www.jacksonfineart.com/exhibitions/83-roger-ballen-boarding-house/`.

2. Yamamoto Masao;. Available from: `https://www.jacksonfineart.com/artists/yamamoto-masao/`.

3. Lalla Essaydi;. Available from: `https://www.jacksonfineart.com/artists/lalla-essaydi/`.

4. Mona Kuhn;. Available from: https://www.jacksonfineart.com/artists/mona-kuhn.
5. Cig Harvey;. Available from: https://www.jacksonfineart.com/artists/cig-harvey/.
6. Epilogue, July 15 - August 27, 2005;. Available from: https://www.jacksonfineart.com/exhibitions/130-epilogue.
7. Angela West;. Available from: https://www.jacksonfineart.com/artists/angela-west.
8. Native by Mona Kuhn;. Available from: https://www.lensculture.com/books/10357-native.
9. Saul Leiter Foundation - Painter and Photographer;. Available from: https://www.saulleiterfoundation.org/.
10. Angela West - Sweet 16, July 8 - August 21, 2004;. Available from: https://www.jacksonfineart.com/exhibitions/135-angela-west-sweet-16/.
11. Holly Andres;. Available from: https://www.jacksonfineart.com/artists/holly-andres/.
12. Jeannette Montgomery Barron: Scene, September 6 - November 2, 2013;. Available from: https://www.jacksonfineart.com/exhibitions/61-jeannette-montgomery-barron-scene/.
13. Bio;. Available from: https://www.angelawest.net/about.
14. Jeannette Montgomery Barron: My mother's clothes, July 29 - August 28, 2010;. Available from: https://www.jacksonfineart.com/exhibitions/86-jeannette-montgomery-barron-my-mother-s-clothes/.
