# Supplementary material for: How deep is your art: An experimental study on the limits of artistic understanding in a single-task, single-modality neural network
Source: PLoS One. 2024 Nov 6;19(11):e0305943. doi: 10.1371/journal.pone.0305943 (PMC11540182; doi:10.1371/journal.pone.0305943)
Supplement: S4 Table — (PDF) [file pone.0305943.s004.pdf]

Table 4. Dataset info: Galleries, Images (number of images per gallery), Total (total number of images per dataset).

Dataset	Gallery	Images	Total
S1	*Heat + High Fashion*	6	64
	Mukono	16	
	My Mother's Clothes	22	
	Scene	13	
	Trigger	7	
S2	*Bullets*	7	47
	Heat + High Fashion	6	
	Painted Nudes	14	
	Persephone	7	
	The Fall of Spring Hill	13	
S3	*Boarding House*	13	72
	My Mother's Clothes	22	
	Painted Nudes	14	
	Private	16	
	Trigger	7	
S4	*Heat + High Fashion*	6	98
	Mukono	16	
	My Mother's Clothes	22	
	Non-Art	34/18	
	Scene	13	
	Trigger	7	
G1	*30 Years of Women*	51	119
	Bullets	7	
	Epilogue	14	
	Paradise Lost	22	
	The Unknown	18	
	Trigger	7	
SF1	*Converging Territories*	7	32
	Heat + High Fashion	6	
	Hivernacle	5	
	Persephone	7	
	Trigger	7	
SF2	*Familiar Landscapes*	10	44
	Little Deaths	9	
	Persephone	7	
	Sweet 16	11	
	Trigger	7	
SF3	*Close*	11	87
	Evidence	17	
	Kawa = Flow	17	
	Native	15	
	The Fall of Spring Hill	13	
	The Fallen Fawn	14	
SF4	*Bonsai*	27	144
	Eat Flowers	25	
	My Mother's Clothes	22	
	New york, Paris, and Rome	32	
	The Garden	20	
	The Unknown	18	
