# Supplementary material for: How deep is your art: An experimental study on the limits of artistic understanding in a single-task, single-modality neural network
Source: PLoS One. 2024 Nov 6;19(11):e0305943. doi: 10.1371/journal.pone.0305943 (PMC11540182; doi:10.1371/journal.pone.0305943)
Supplement: S5 Table — (PDF) [file pone.0305943.s005.pdf]

Table 5. Numerical values of statistical tests and measures.

Dataset	Max	Min	STD	Mean	Median	SW_p	Leven's	DT3	G-H	Tukey's
**S1**	1.000	0.375	0.101	0.747	0.750	< 0.001	< 0.001	< 0.001	< 0.001	< 0.001
**Difficult**	0.938	0.188	0.108	0.677	0.688	< 0.001	< 0.001	0.374	0.283	0.236
**Average**	1.000	0.313	0.115	0.684	0.688	< 0.001	< 0.001	0.374	0.283	0.236
**Easy**	1.000	0.313	0.097	0.798	0.813	< 0.001	< 0.001	< 0.001	< 0.001	< 0.001
**S2**	1.000	0.333	0.087	0.931	0.917	< 0.001	< 0.001	< 0.001	< 0.001	< 0.001
**Difficult**	1.000	0.250	0.126	0.825	0.833	< 0.001	< 0.001	< 0.001	< 0.001	< 0.001
**Average**	1.000	0.364	0.069	0.952	1.000	< 0.001	< 0.001	< 0.001	< 0.001	< 0.001
**Easy**	1.000	0.583	0.032	0.991	1.000	< 0.001	< 0.001	< 0.001	< 0.001	< 0.001
**S3**	1.000	0.294	0.106	0.774	0.765	< 0.001	< 0.001	< 0.001	< 0.001	< 0.001
**Difficult**	0.824	0.294	0.079	0.617	0.647	< 0.001	< 0.001	< 0.001	< 0.001	< 0.001
**Average**	0.941	0.353	0.081	0.738	0.765	< 0.001	< 0.001	< 0.001	< 0.001	< 0.001
**Easy**	1.000	0.294	0.113	0.860	0.882	< 0.001	< 0.001	< 0.001	< 0.001	< 0.001
**G1**	0.926	0.259	0.094	0.646	0.667	< 0.001	< 0.001	< 0.001	< 0.001	< 0.001
**Difficult**	0.630	0.222	0.065	0.456	0.444	< 0.001	< 0.001	< 0.001	< 0.001	< 0.001
**Average**	0.889	0.333	0.082	0.660	0.667	< 0.001	< 0.001	< 0.001	< 0.001	< 0.001
**Easy**	0.963	0.222	0.116	0.713	0.741	< 0.001	< 0.001	< 0.001	< 0.001	< 0.001
**S4**	0.913	0.217	0.104	0.628	0.652	< 0.001	< 0.001	0.002	0.002	0.001
**Difficult**	0.696	0.174	0.075	0.486	0.478	< 0.001	< 0.001	< 0.001	< 0.001	< 0.001
**Average**	0.870	0.261	0.098	0.642	0.652	< 0.001	< 0.001	0.002	0.002	0.001
**Easy**	0.957	0.304	0.107	0.681	0.696	< 0.001	< 0.001	< 0.001	< 0.001	< 0.001
**SF1**	1.000	0.333	0.132	0.821	0.778	< 0.001	< 0.001	0.162	0.128	0.141
**SF2**	1.000	0.182	0.117	0.729	0.727	< 0.001	< 0.001	< 0.001	< 0.001	< 0.001
**SF3**	0.850	0.150	0.103	0.550	0.550	< 0.001	< 0.001	< 0.001	< 0.001	< 0.001
**SF4**	1.000	0.419	0.070	0.829	0.839	< 0.001	< 0.001	0.162	0.128	0.141
