# Supplementary figures and images for: How deep is your art: An experimental study on the limits of artistic understanding in a single-task, single-modality neural network
Source: PLoS One. 2024 Nov 6;19(11):e0305943. doi: 10.1371/journal.pone.0305943 (PMC11540182; doi:10.1371/journal.pone.0305943)

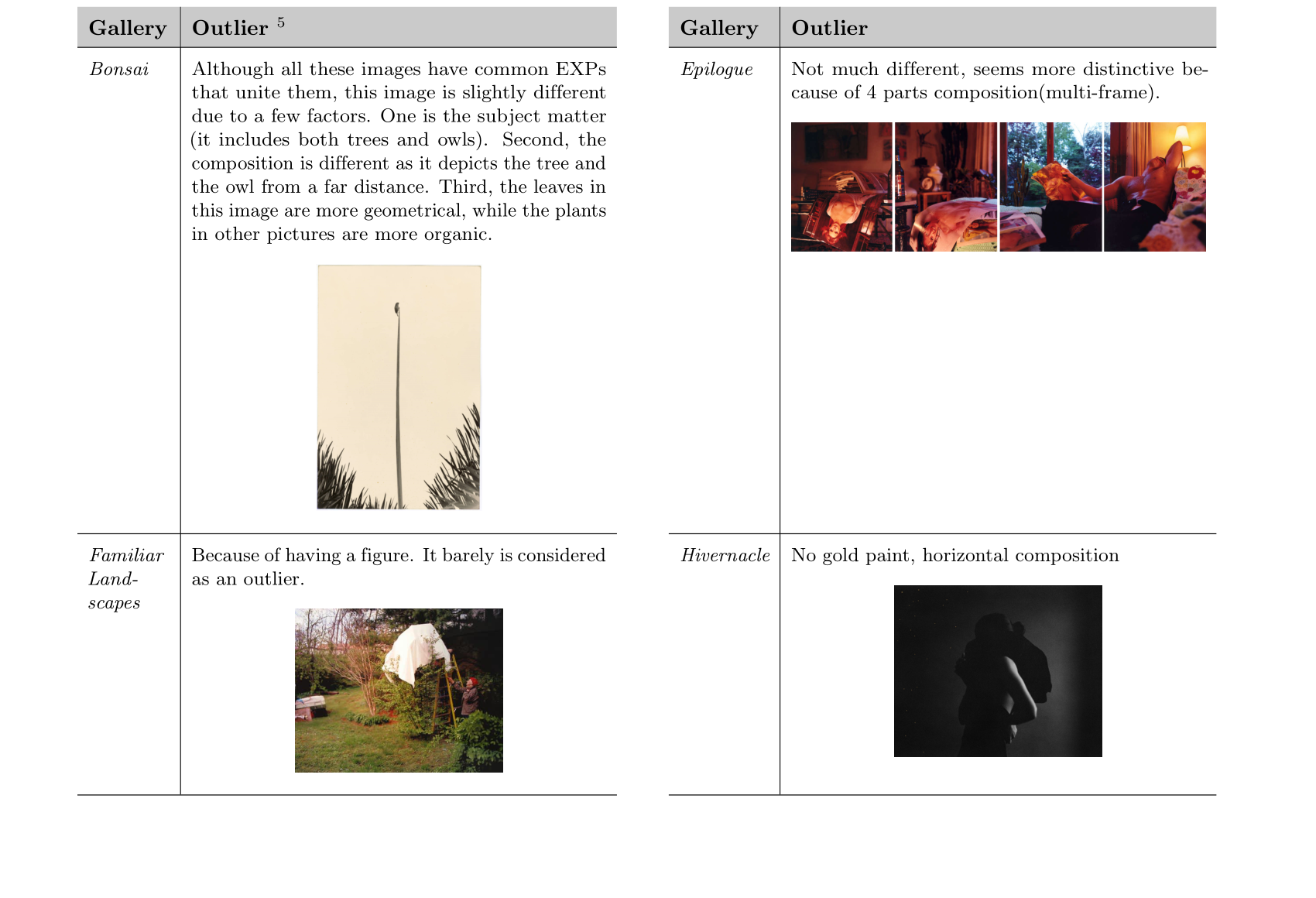

Supplement: S1 Fig — (TIF) [file pone.0305943.s006.tif]

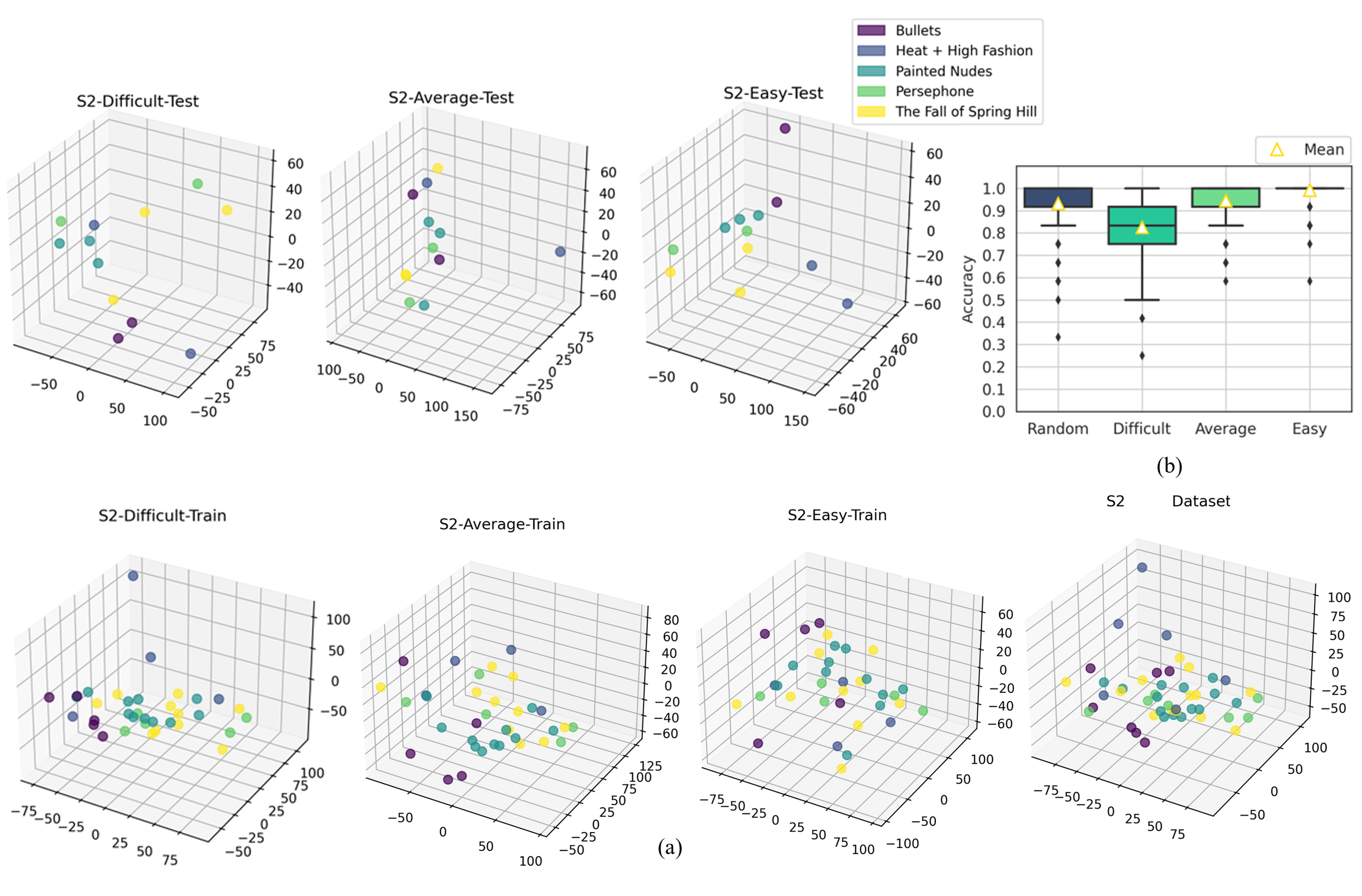

Supplement: S2 Fig — (a) PCA plots for the training and test data of subsets difficult, average, and easy. (b) Box and whisker plots of the overall accuracies (ACC) of the DCNN classification of the four subsets. (TIF) [file pone.0305943.s007.tif]

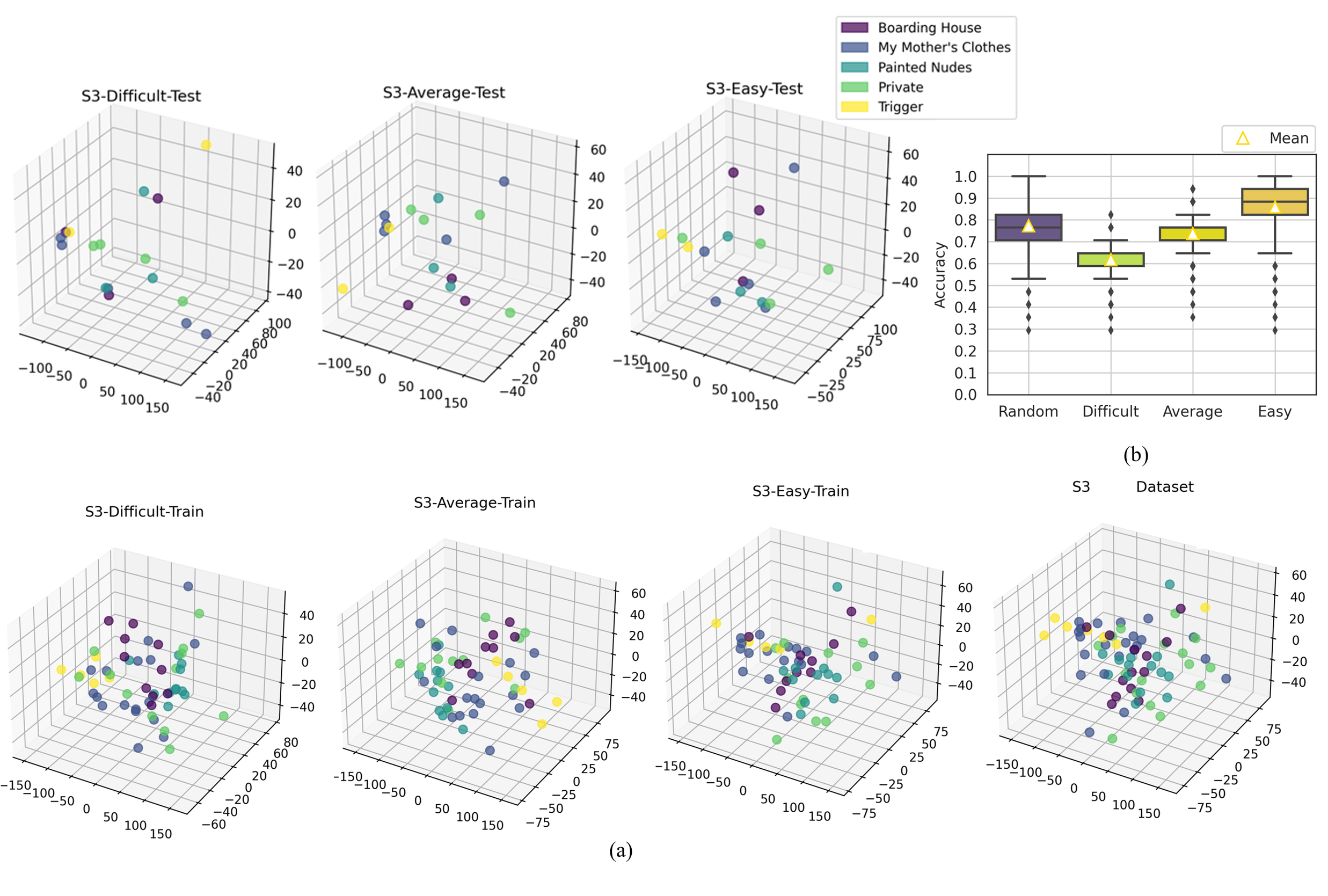

Supplement: S3 Fig — (a) PCA plots for the test sets. (b) Box and whisker plots of the overall accuracies. (TIF) [file pone.0305943.s008.tif]

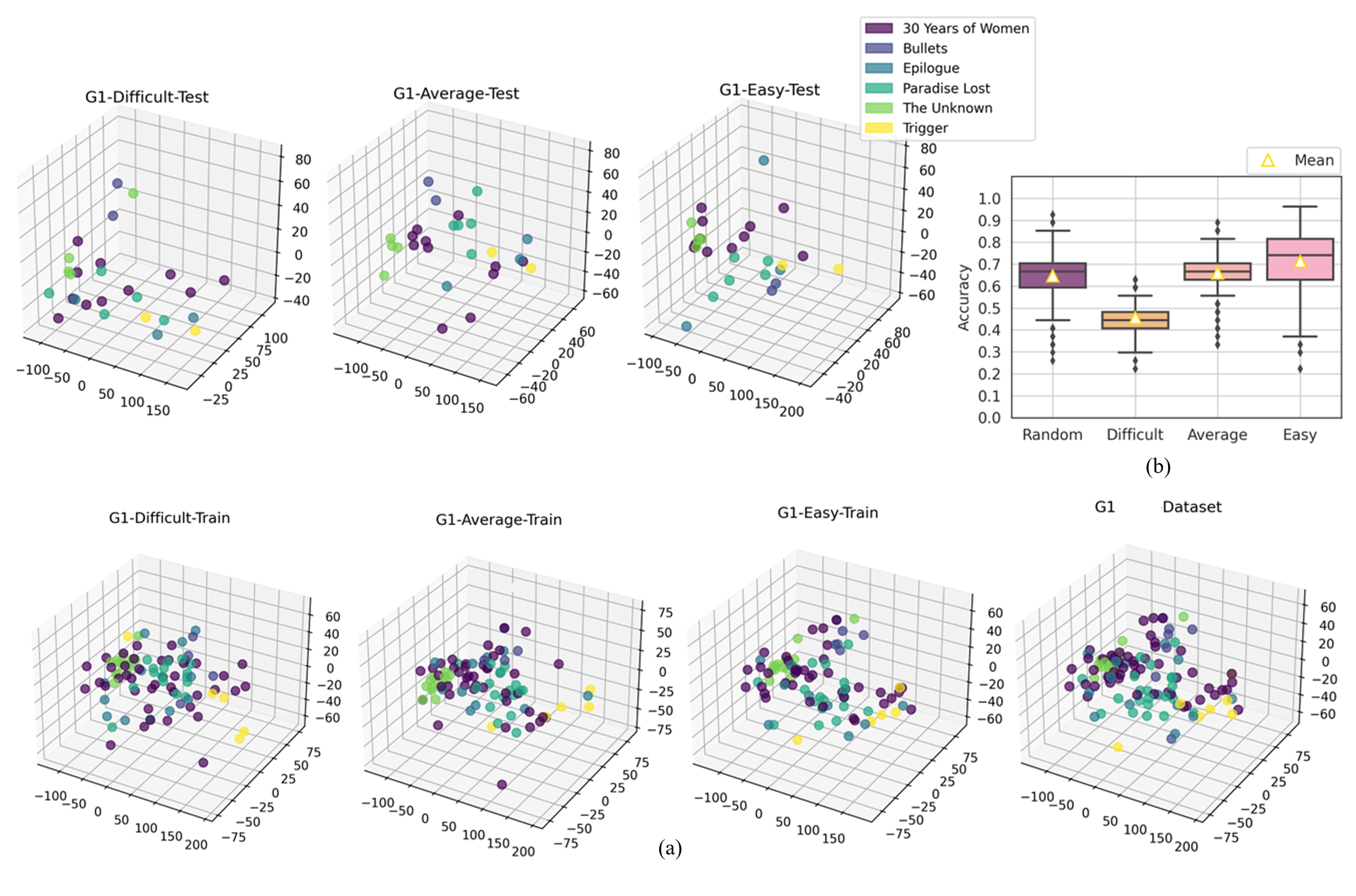

Supplement: S4 Fig — (a) PCA plots for test data for subsets difficult, average, and easy. (b) Box and whisker plot of overall accuracies (ACC) of the DCNN classification of the four subsets. (TIF) [file pone.0305943.s009.tif]

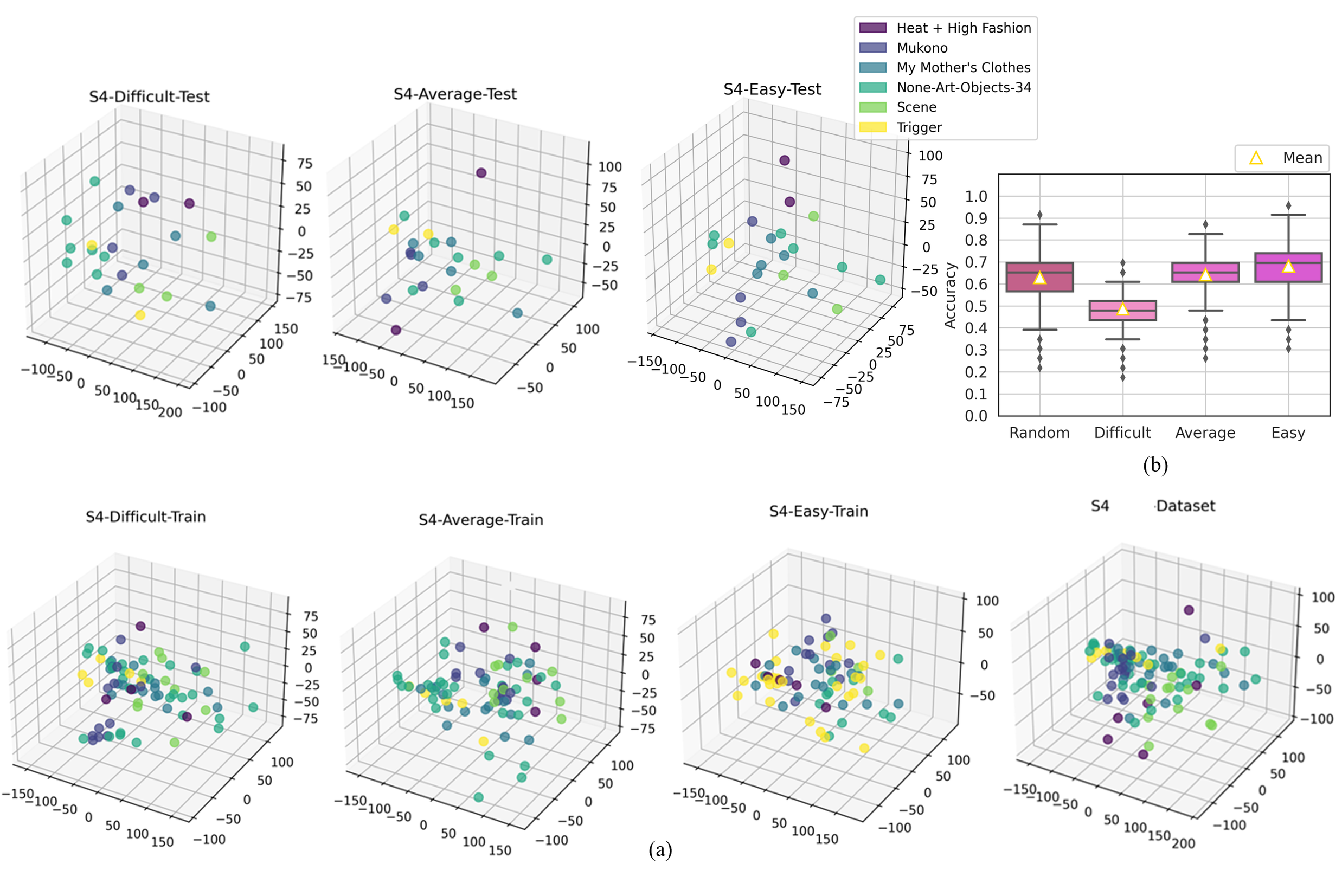

Supplement: S5 Fig — (a) The PCA plots for the training and test sets for subsets difficult, average, and easy. (b) The box and whisker plot of the overall accuracies (ACC) of the DCNN classification of the four subsets. (TIF) [file pone.0305943.s010.tif]

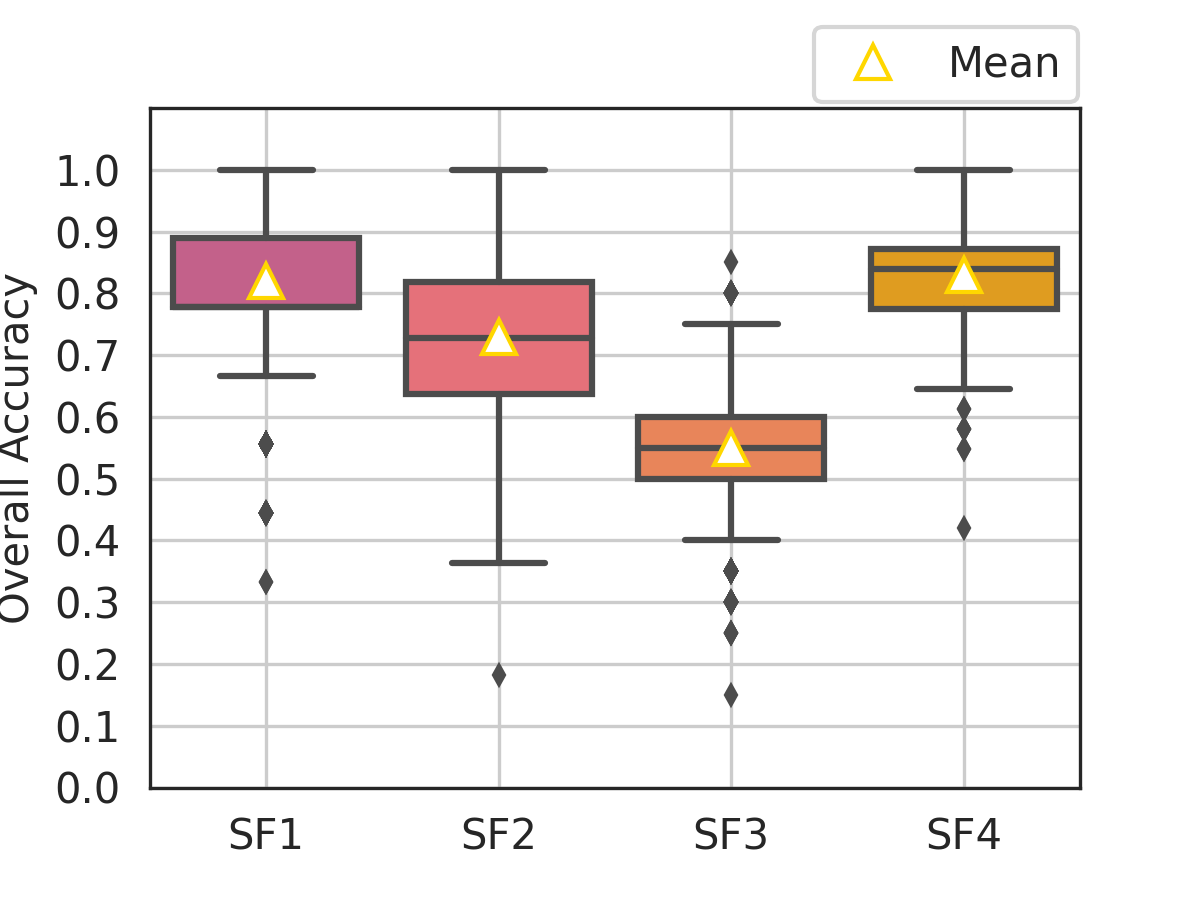

Supplement: S6 Fig — (TIF) [file pone.0305943.s011.tif]

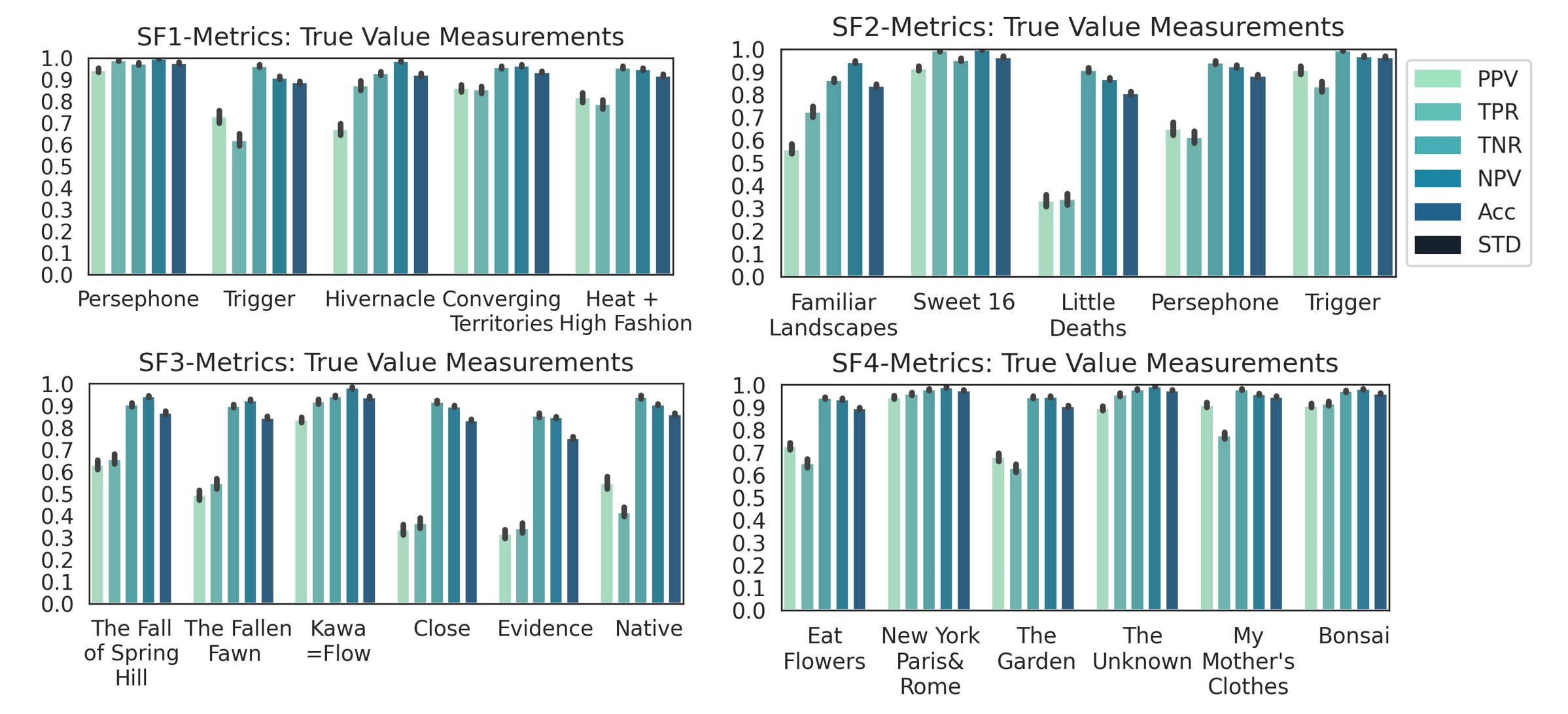

Supplement: S7 Fig — (TIF) [file pone.0305943.s012.tif]

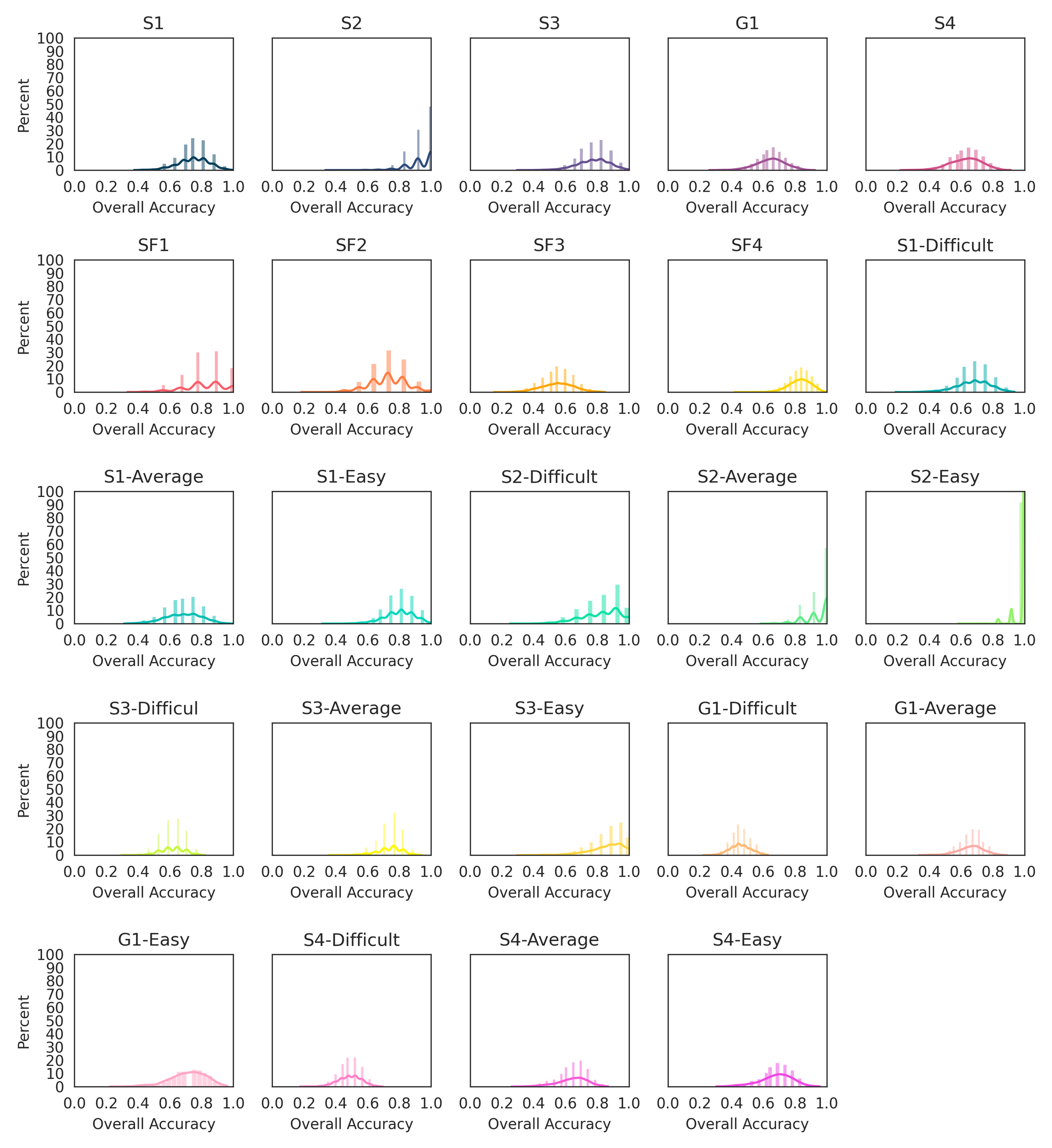

Supplement: S8 Fig — Histogram plots verify the distribution of the measured random variable. (TIF) [file pone.0305943.s013.tif]

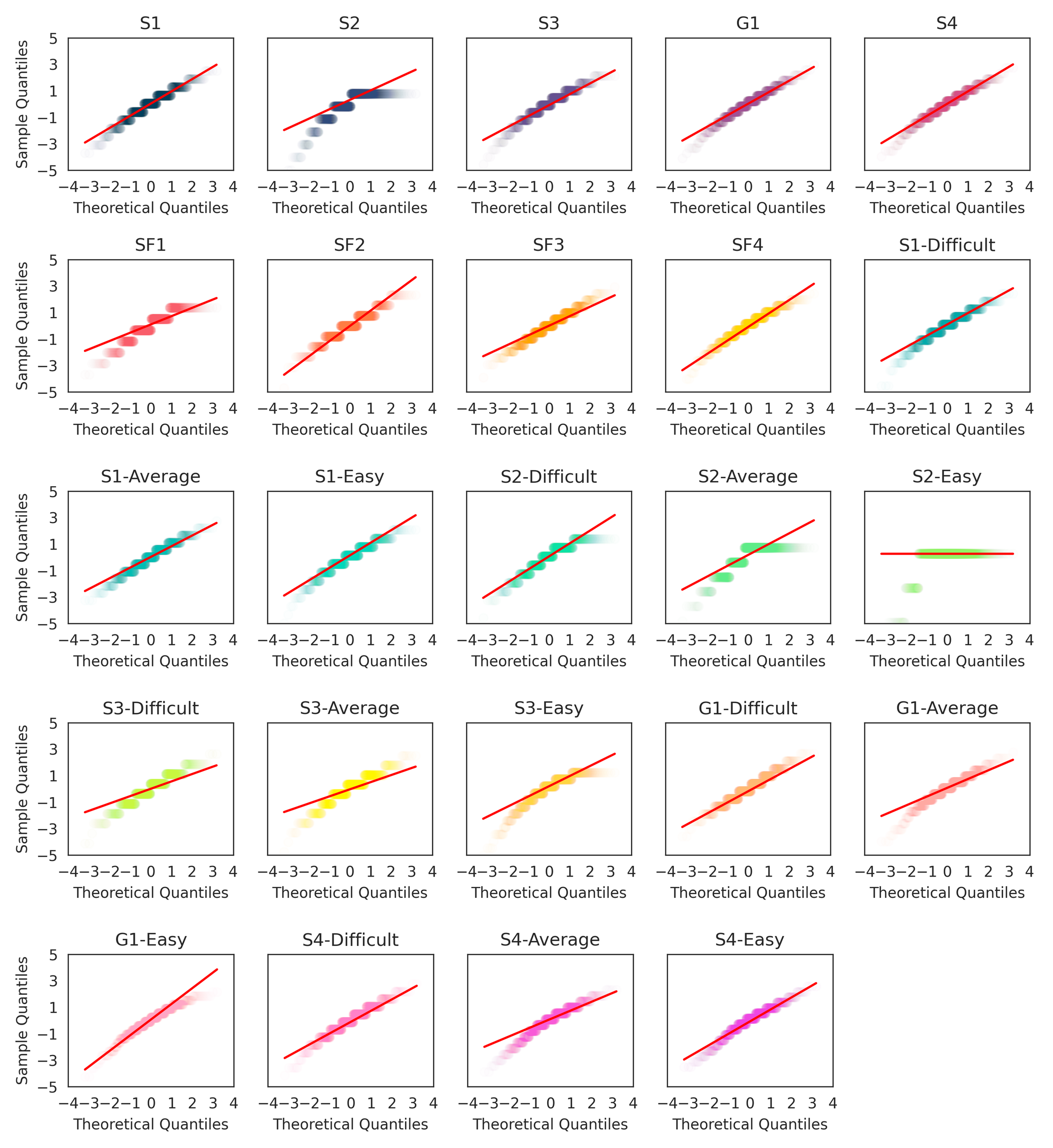

Supplement: S9 Fig — Normal Q-Q plots are another method for verifying the distribution of the measured random variable. (TIF) [file pone.0305943.s014.tif]

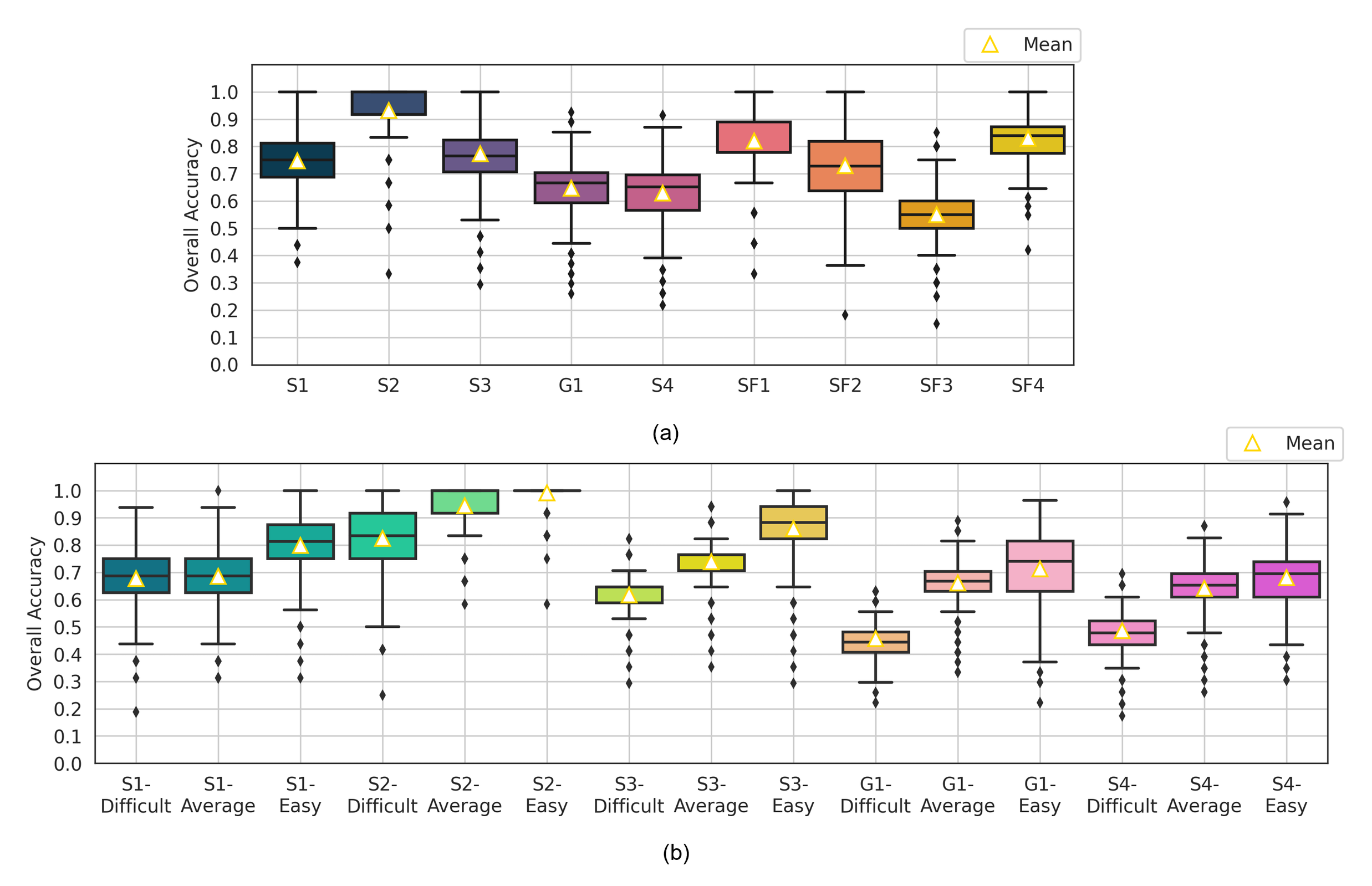

Supplement: S10 Fig — (a) Box and whisker plot of overall accuracies for all datasets random sets (b) Box and whisker plot of overall accuracies for all datasets handpicked sets (difficult, average, and easy). (TIF) [file pone.0305943.s015.tif]

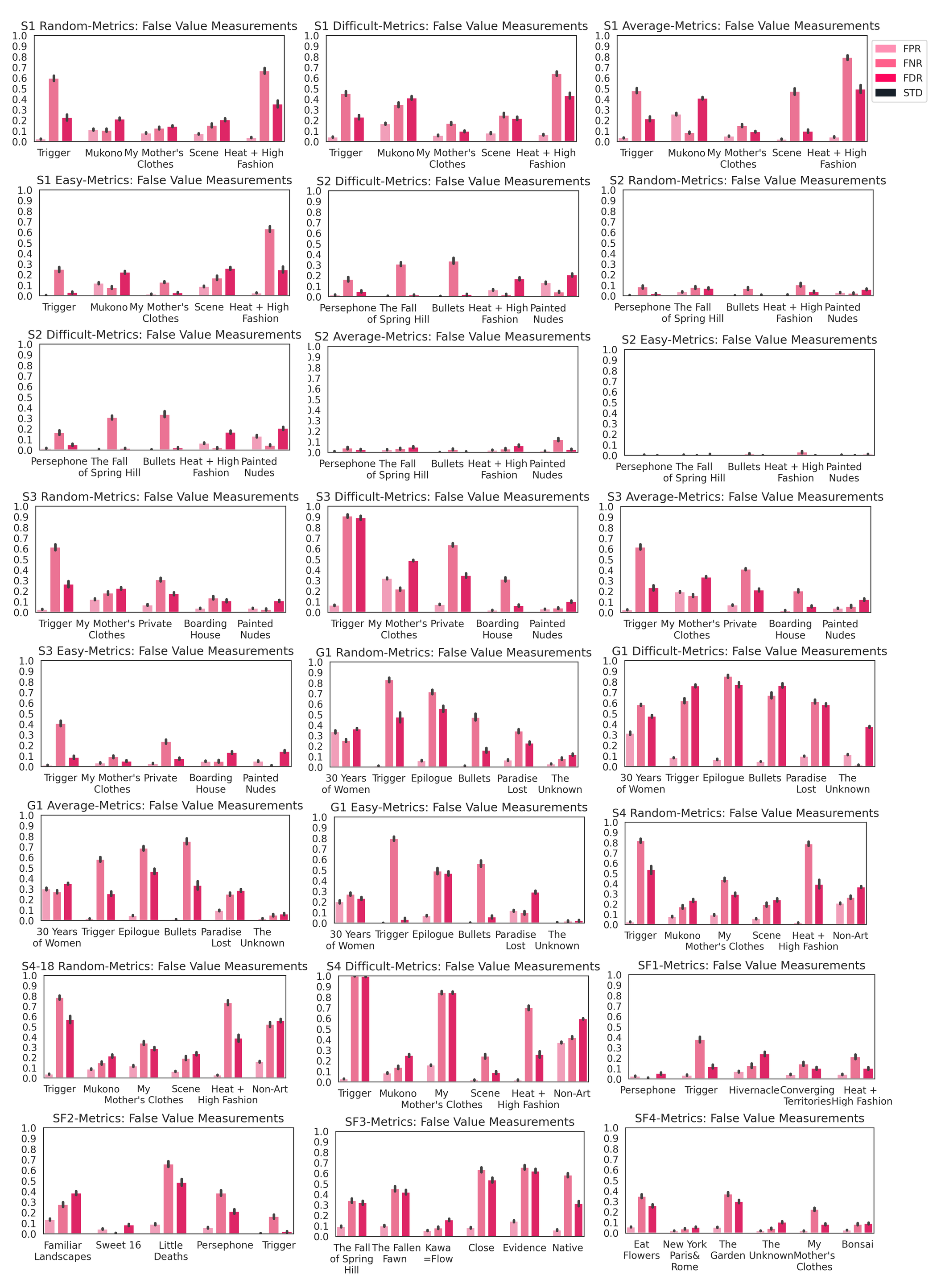

Supplement: S11 Fig — (TIF) [file pone.0305943.s016.tif]
