# Supplementary material for: How deep is your art: An experimental study on the limits of artistic understanding in a single-task, single-modality neural network
Source: PLoS One. 2024 Nov 6;19(11):e0305943. doi: 10.1371/journal.pone.0305943 (PMC11540182; doi:10.1371/journal.pone.0305943)
Supplement: Attachment — Submitted filename: ResponsetoPLoSReviewer.pdf [file pone.0305943.s017.pdf]

Response to the Reviewer's Comments

Acknowledgement: The authors thank the Associate Editor and the reviewer for their comments and feedback. We carefully addressed each of them. We also rewrote the paper to simplify the presentation and make the description more rigorous.

Please find bellow our responses to the received comments and suggestions.

Comment: There is little or no mention of the substantial literature that relates aesthetic decision to sensory coding (see for example Redies, C. (2015). Frontiers in Human Neuroscience

Response: Thank you for pointing out this work. We added additional references to address the reviewer's comment. Lines 38-43, 102-104, and 117-120 in the revision discuss the added references.

Comment: The phrase "meaning. Previous work suggests that DNN models can gain some insight on meaning (semantics) starting only from EXPs, like color, texture, and shapes" needs references

Response: The references were added to the indicated sentence.

Comment: The phrase in L167-168: "The ambiguity of two galleries is higher if there are less common EXPs that refer to the same NEXPs, or if more common EXPs relate to the same NEXPs." At some level of interpretation it seems to contradict itself and is also difficult to understand. Please clarify.

Response: We rewrote the paragraph to explain the used definition of a gallery's classification difficulty. The new description is between lines 183-197, and states as follows:

"A dataset was considered to be more difficult to classify, if its images from multiple galleries (with different themes, and hence likely with distinct NEXPs) were more similar with respect to their EXPs, or if its images in a gallery had a high diversity of EXPs. For instance, two galleries that contain black and white photography look more similar with respect to their EXPs (e.g., their colors). If the two hypotheses are correct then this similarity makes it harder for the DCNN model to classify the images into their correct gallery, even if these galleries have different NEXPs, i.e. due to their different historical context and meaning. Moreover, a high diversity of EXPs within a single gallery increases the classification difficulty because it is harder for the DCNN model to find common EXPs to identify the images that belong to the same gallery, even if the gallery's artworks have similar NEXPs. This observation is especially evident for group exhibitions, since they were curated around similar themes and meanings but they contain artwork that looks

different from each other as distinct artists created them. Hence, separate datasets studied the using of EXPs and NEXPs in solo and group galleries for classification."

Comment: The reasons for choices of experiments (L82-84) are not clear and should be explained in detail.

Response: The constructed datasets included photographs that represent contemporary photography in its diversity of style and conceptual orientation. Our art expert chose exhibitions of artists from different countries and photographs that reflect different approaches toward fine art photography, like realism, abstracted, commercial, and conceptual art. As discussed in Section Introduction, conceptual art such as Duchamp's *Urinal* often represents ordinary, ready-made or mass-produced objects as a work of art, relying on the ideas behind it. We predicted that conceptual photography would particularly increase the level of difficulty of the dataset. To further experiment with conceptual photography, and particularly to experiment with how much the model can move beyond the EXPs and classify the art in terms of their NEXs, meaning concept, ideas, and historical context, we included a gallery of none-art photographs of ordinary objects that in their appearance, resembled the conceptual fine art photography exhibitions included in the experiment. In other words, the images in the non-art gallery *looked* like the photos in the conceptual photography exhibition, though they did not belong to the same categories, nor were they fine arts. We predicted that the DCNN model would show a poor performance in the data set that included conceptual photography and the non-art gallery because of the high number of similarities of EXPs among the galleries.

Subsection "Dataset design" (lines 175 – 229) was entirely rewritten to simply the presentation. Lines 211 – 222 present the above explanation as follows: "The considered art galleries were chosen from existing online exhibitions curated by established art curators, and not by the art expert that participated to our study. To limit the scope of the datasets, we picked artwork that uses photography as an underlying medium, so that the galleries reflect diverse approaches towards fine arts photography in contemporary art. The artwork pursues different artistic attitudes, like highly conceptual, representational, and abstract. Certain art objects mix photography with paint, some used digital, and others are analog photography. We also included group exhibitions, as group exhibitions are curated around a common theme or concept but are vastly different in their styles. Table 1 summarizes the characteristics of the galleries in Dataset S1 (see the Appendix for the other datasets). Table 2 details the EXPs and Table 3 the NEXPs of the galleries in this dataset. Table 4 shows the outliersof the galleries, such as the images that are different from the rest."

Comment: I could not find basic information like the number of images in each of the datasets/galleries in the main paper. There is some information in the supplement, but its presence is inconsistent.

Response: Subsection "Dataset Design" was modified to include the characteristics of Dataset S1. The characteristics of the other datasets used in the experiments were provided in the Supplementary documents. The following four tables were added to Subsection "Dataset Design"

about Dataset S1: Table 1 indicates the characteristics of the Dataset S1. Table 2 enumerates the EXPs of the galleries in Dataset S1. Table 3 presents the NEXPs of the galleries of Dataset S1. Table 4 shows the outliers of Dataset S1.

Comment: More information is needed on how the art expert designed these galleries to meet the purpose of each experiment. Are these themes common in the art world? Were decided on the bases of images availability? Were these galleries provided with the same number of samples? How many galleries? How many pictures are in each of them? Were the galleries the same in all experiments?

Response: Please see our previous two answers for this comment. Also, the art galleries are chosen from existing online exhibitions mainly from Jackson Fine Art. Our art expert did not design the galleries individually but chose them from the already curated online exhibitions and put them together to design the data sets. To establish some limitations on the scope of the research's data sets, we chose artwork that engages photography as an underlying medium, yet the galleries reflect different ways of using photography as a medium. Some mix photography with paint, some use digital and others analog. The work is also diverse in terms of their artistic attitudes; some are highly conceptual, some representational and some more abstract. Also, we chose exhibitions that have different conceptual dimensions and ideas while making sure they represent the diversity of approach toward photography in contemporary art, where many times the ideas and stories behind the photograph is critical to understand the work. In this respect, contemporary art is particularly different from older art movements such as impressionism, expressionism, etc. when stylistic analysis could easily bring us close to understanding and interpreting the works of art. We also made sure to include group exhibitions, as group exhibitions are usually curated around a common theme or concept but are vastly different in terms of their style. Hence, the themes in the exhibitions, therefore, could reflect common themes and approaches toward art and particularly photography in the art world in a cross-cultural scope.

Lines 175-229 in Subsection "Dataset Design" indicate how the galleries were selected, their size, the gallery number in each dataset. Different datasets shared some galleries but also included new galleries depending on the dataset's role in the experiments. Tables 1- 4 summarize the main characteristics, EXPs, NEXPs, and outliers of Dataset S1.

Comment: The information about the different datasets and, in particular, the galleries is sparse, confusing and incomplete. I have tried to understand their properties by summarizing them in a table (see below) but some information is lacking. The description of the datasets in the appendix is somewhat confusing and insufficient. Please clarify.

Response: Thank you very much for your feedback. It was extremely useful to us to redo Subsection "Dataset Design". We added Tables 1-4 to the subsection. These tables follow the concept that you suggested in your feedback.

Comment: The words "experiment" and "setting" are sometimes used interchangeably. Should stick to a single meaning/definition.

Response: We revised the entire document to address the comment.

Comment: What Type of data augmentation was used?

Response: The following description was added as lines 250-252: “To avoid overfitting, batch normalization [26] was used as a regularization technique along with data augmentation using methods Random Rotation, Random Horizontal Flip, and Random Crop with Padding from Torchvision library.”

```
train_transforms = transforms.Compose([
                        transforms.Resize(pretrained_size),
                        transforms.RandomRotation(5),
                        transforms.RandomHorizontalFlip(0.5),
                        transforms.RandomCrop(pretrained_size, padding = 10),
                        transforms.ToTensor(),
                        transforms.Normalize(mean = pretrained_means,
                                             std = pretrained_stds)
                    ])
```

Comment: I did not understand the information displayed in the PCA plots. There is a brief description in L218-226 but it does not make much sense for me: The PCAs were applied to pixels in colour space (which colour space? sRGB? what happened to grayscale images?) and there is no information on how the information for all large groups of images was aggregated (the explanation only talks about a single image providing 150,528 datapoints). The authors need to explain how were these clusters (Figures 5,7,9,11 and 13) obtained. Also, I doubt all of them were the same size, therefore, some indication of their sizes (and overlapping) should be provided. Please explain the PCA procedure in full....

Response: Principal Component Analysis (PCA) was used to visualize the designed datasets for further verification with respect to their expected levels of difficulty. Each image in a dataset underwent the same preprocessing that was also used for DCNN model, except for data augmentation transformations that were resized and center-cropped to a 224 × 224-pixel image in RGB color space.

Lines 238-246 in Subsection “Design Verification” present this information.

The first three principal components of each image were plotted in a 3D scatter plot, where each gallery is illustrated by a separate color. The formation of clusters with points of the same colors indicates a successful distinguishing of the images of a gallery using the EXPs of that gallery. NEXPs are not considered by PCA.

Lines 299-302, 382-386, 427-229, 472 – 474, and 523-525 refer to the using of PCA for dataset validation and have been changed to address the reviewer's comments.

```
_transforms = transforms.Compose([
                                  transforms.Resize(pretrained_size),
                                  transforms.CenterCrop(pretrained_size),
                                  transforms.ToTensor()]#,
                                  #transforms.Normalize(mean = pretrained_means,
                                                       #std = pretrained_stds)]
                                  )
```

Comment: I do not understand the phrase "As the experiments section explains, PCA results did not depict full clusters due to the fact that NEXPs were not represented".

Comment: I found the description of the different experiments, parameters, galleries and settings confusing and poorly organised. I recommend that the authors review this in full to make the paper easy to follow.

Response: We rewrote the entire document to improve its presentation, make it better organized, and eliminate repetitive descriptions in the document. We hope that the revision addresses the reviewer's comment.

Minors:
L68: where it says "...which might not visible..." should say "...which might not be visible..."
The labels in Figure 3(a) say "none exhibited properties" where it should say "non-exhibited properties"

Response: The mentioned typos were corrected.
