# Supplementary material for: How deep is your art: An experimental study on the limits of artistic understanding in a single-task, single-modality neural network
Source: PLoS One. 2024 Nov 6;19(11):e0305943. doi: 10.1371/journal.pone.0305943 (PMC11540182; doi:10.1371/journal.pone.0305943)
Supplement: Attachment — Submitted filename: PLOS_ONE_rebuttal_05_10_2024.pdf [file pone.0305943.s018.pdf]

PLOS ONE
1265 Battery Street, Suite 200
San Francisco, CA 94111
United States
+1 415 624 1200

Mahan Agha Zahedi
Electrical and Computer Engineering
Stony Brook University
Stony Brook NY 11794-2350
United States
Mahan.AghaZahedi@stonybrook.edu
+1 914-619-4767

Dear Academic Editor,

Please find hereby the revised version of our manuscript *PONE-D-23-31505* titled, "*How deep is your art: an experimental study on the limits of artistic understanding in a single-task, single-modality neural network*", which we originally submitted for consideration to PLOS ONE.

We would like to express our gratitude to the reviewers and the editorial team for their constructive feedback, and critical assessment which has undoubtedly helped enhance the quality of our work. We appreciate the time and effort invested in the process.

In this revised manuscript, we have diligently addressed all the concerns raised by the editorial team and the reviewers to the best of our abilities. We have provided a point-by-point response in the following section, *Response to the Reviewers*. We believe that these revisions have significantly strengthened the manuscript and improved its overall clarity and impact. We hope that the revised version meets the rigorous standards of PLOS ONE and will make a valuable contribution to the scientific community.

The changes that addressed the reviewers' comments were highlighted in yellow in the revision. In addition to these, some sentences were re-written throughout the entire paper to improve the paper structure and presentation.

Thank you for considering our revised manuscript for publication in PLOS ONE. We look forward to hearing from you soon.

Sincerely Yours,

Mahan Agha Zahedi (He/His/Him)
Contact Person

Response to the reviewers

Journal Requirements

1. *Please ensure that your manuscript meets PLOS ONE's style requirements, including those for file naming. The PLOS ONE style templates can be found at* `https://journals.plos.org/plosone/s/file?id=wjVg/PLOSOne_formatting_sample_main_body.pdf` *and* `https://journals.plos.org/plosone/s/file?id=ba62/PLOSOne_formatting_sample_title_authors_affiliations.pdf`

Response:

We have ensured that our revised manuscript and its files meet PLOS ONE's style requirements.

2. *Note from Emily Chenette, Editor in Chief of PLOS ONE, and Iain Hrynaszkiewicz, Director of Open Research Solutions at PLOS:*
Did you know that depositing data in a repository is associated with up to a 25% citation advantage (`https://doi.org/10.1371/journal.pone.0230416`*)? If you've not already done so, consider depositing your raw data in a repository to ensure your work is read, appreciated and cited by the largest possible audience. You'll also earn an Accessible Data icon on your published paper if you deposit your data in any participating repository (*`https://plos.org/open-science/open-data/#accessible-data`*).*

Response:

We appreciate this valuable note to make our dataset and code publicly available. The code was made available as requested.

However, for copyright-related reasons, we could not accommodate the request about the datasets (e.g., to make the images available to the public via a donwloadable file) but we ensured that our datasets are accessible to the reviewers via the following Google Drive link, `https://drive.google.com/drive/folders/1oe8SalgoVpREfjgdEUW6tSTc1XtL8rYV?usp=sharing`, to facilitate the review process.

In compliance with the U.S. Office of Copyright Fair Use, we were able to use copyrighted material for this research study. Our response to point (7) further explains this in detail. However, we cannot distribute the dataset as it will breach the *Amount and Substantiality of the Portion Used*, as we will upload the images in full scale and resolution.

However, Table 5 in Supporting Information contains all the necessary information, including the links to the images, to enable anyone interested to reconstruct the datasets on their own. We tried to address this requirement to the best of our abilities without infringing the copyright rules.

3. *Please note that PLOS ONE has specific guidelines on code sharing for submissions in which author-generated code underpins the findings in the manuscript. In these cases, all author-generated code must be made available without restrictions upon publication of the work.*
Please review our guidelines at `https://journals.plos.org/plosone/s/materials-and-software-sharing#loc-sharing-code` *and ensure that your code is shared in a way that follows best practice and facilitates reproducibility and reuse.*

Response:

The code implementation and the information to reconstruct the datasets are open-accessed via the following link on GitHub.

`https://github.com/aghazahedim/How-Deep-is-Your-Art/tree/main`

4. *In the online submission form, you indicated that: "Datasets cannot be shared publicly because of copyright. However, we can provide the datasets upon request for academic/non profit purposes according to fair use act."*

   All PLOS journals now require all data underlying the findings described in their manuscript to be freely available to other researchers, either

   (a) *In a public repository,*

   (b) *Within the manuscript itself, or*

   (c) *Uploaded as supplementary information.*

   This policy applies to all data except where public deposition would breach compliance with the protocol approved by your research ethics board. If your data cannot be made publicly available for ethical or legal reasons (e.g., public availability would compromise patient privacy), please explain your reasons on resubmission and your exemption request will be escalated for approval.

Response:

The code implementation and the information needed to reconstruct the datasets were provided and are open-accessed via the following link on GitHub and DOI: 10.5281/zenodo.11167490.

`https://github.com/aghazahedim/How-Deep-is-Your-Art/tree/main`

5. *Please amend the manuscript submission data (via Edit Submission) to include authors:*
   - Niloofar Gholamrezaei
   - Alex Doboli

Response:

This has been resolved in the resubmission.

6. *Please upload a copy of Figure 16, to which you refer in your text on page 23. If the figure is no longer to be included as part of the submission please remove all reference to it within the text.*

Response:

This has been resolved in the revised manuscript.

7. *We note that Figures and Supporting Figures in your submission contain copyrighted images:*
   - *Fig_1*
   - *Fig_4*
   - *Table_4_Mukono*
   - *Table_4_Trigger*
   - *S2_Table_4_Bonsai*
   - *S2_Table_4_Epilogue*
   - *S2_Table_4_Familiar_Landscapes*
   - *S2_Table_4_Hivernacle*
   - *S2_Table_4_Little_Deaths*
   - *S2_Table_4_Mukono*
   - *S2_Table_4_Native*
   - *S2_Table_4_Paradise_Lost*
   - *S2_Table_4_Private*

- *S2_Table_4_The_Fall_of_Spring_Hill*
- *S2_Table_4_The_Fallen_Fawn*
- *S2_Table_4_Trigger*

All PLOS content is published under the Creative Commons Attribution License (CC BY 4.0), which means that the manuscript, images, and Supporting Information files will be freely available online, and any third party is permitted to access, download, copy, distribute, and use these materials in any way, even commercially, with proper attribution. For more information, see our copyright guidelines: `http://journals.plos.org/plosone/s/licenses-and-copyright`*.*
We require you to either (1) present written permission from the copyright holder to publish these figures specifically under the CC BY 4.0 license, or (2) remove the figures from your submission:
(1) You may seek permission from the original copyright holder of those figures & supporting figures mentioned above to publish the content specifically under the CC BY 4.0 license.
We recommend that you contact the original copyright holder with the Content Permission Form (`http://journals.plos.org/plosone/s/file?id=7c09/content-permission-form.pdf`*) and the following text:*
"I request permission for the open-access journal PLOS ONE to publish XXX under the Creative Commons Attribution License (CCAL) CC BY 4.0 (`http://creativecommons.org/licenses/by/4.0/`*). Please be aware that this license allows unrestricted use and distribution, even commercially, by third parties. Please reply and provide explicit written permission to publish XXX under a CC BY license and complete the attached form."*
Please upload the completed Content Permission Form or other proof of granted permissions as an ""Other"" file with your submission.
In the figure caption of the copyrighted figure, please include the following text: "Reprinted from [ref] under a CC BY license, with permission from [name of publisher], original copyright [original copyright year]."
(2) If you are unable to obtain permission from the original copyright holder to publish these figures under the CC BY 4.0 license or if the copyright holder's requirements are incompatible with the CC BY 4.0 license, please either i) remove the figure or ii) supply a replacement figure that complies with the CC BY 4.0 license. Please check copyright information on all replacement figures and update the figure caption with source information.
If applicable, please specify in the figure caption text when a figure is similar but not identical to the original image and is therefore for illustrative purposes only.

Response:

Fig_1, contains Marcel Duchamp's (1887-1968) "Fountain" and William Geets'(1838-1919) "Accident". According to U.S. Copyright Office, for works created before 1978, the copyright term is the life of the author plus 70 years. Therefore, the copyright for "Fountain" expired at the end of 2018, making it public domain as of January 1, 2019. Similarly, Since we are beyond the 70-year threshold after Geets' death, "Accident" and all other works by Geets entered the public domain as of January 1, 1990. Thus, both of these works are now in the public domain, and can be used freely by anyone without needing permission for reproduction, distribution, or creation of derivative works.

`https://www.copyright.gov/help/faq/faq-duration.html`
`https://www.copyright.gov/circs/circ15a.pdf`

The rest of the figures, Fig_4 to S2_Table_4_Trigger, contain copyrighted photography pieces from Jackson Fine Art Gallery. According to the fair use doctrine terms, our usage complies with the four statutory factors of fair use. These factors are outlined below:

1. **Purpose and Character of the Use:**
   The use for academic and non-profit purposes supports fair use.

2. **Nature of the Copyrighted Work:**
   The use in an academic paper, particularly adds scholarly and educational value, thus, it is considered transformative, adding further support for fair use.

3. **Amount and Substantiality of the Portion Used:**
   We have used lower resolution and smaller image sizes, which is favorable for fair use, and suggests that only the amount necessary for educational purposes is being used.

4. **Effect on the Market:**
   The publication of your academic paper does not replace the market for the original photographs, especially if used in a way that does not satisfy the same need as the original works (such as display or sale of high-quality prints). Hence, it does not harm the sales or value of the original photographs. On the contrary, the use of the images in this manuscript could potentially draw more attention to the original works providing more visibility to these artworks.

"*Fair use is a legal doctrine that promotes freedom of expression by permitting the unlicensed use of copyright-protected works in certain circumstances.*"
`https://www.copyright.gov/fair-use/`

8. *We notice that your supplementary figures (Table_4_Mukono and Table_4_Trigger) are uploaded with the file type 'Figure'. Please remove the file and leave only the supplementary figures (S2_Table_4_Mukono and S2_Table_4_Trigger) uploaded. Please ensure that each Supporting Information file has a legend listed in the manuscript after the references list.*

Response:

This has been resolved in the resubmission.

9. *Please include captions for your Supporting Information files at the end of your manuscript, and update any in-text citations to match accordingly. Please see our Supporting Information guidelines for more information:* `http://journals.plos.org/plosone/s/supporting-information`.

Response:

This has been resolved in the resubmission.

Reviewers' comments:

5. Review Comments to the Author

   Reviewer #1:

   - *However, the innovations in this paper focus on transitional convolution neural network, and the methods and theoretical innovations are not sufficient. The author must dig deeper into the innovation points before the article can be accepted by PONE.*

Response:

The reviewer's comment was addressed as follows in the revised manuscript. The changes were highlighted in yellow in the document.

We added the following descriptions to the paper to address this comment. The paragraph at the bottom of Page 4 starting with "A novel experimental method ..." was added to explain that a new experimental method was devised in this work to ensure that the testing of the two hypotheses proceeded in an systematic

and consistent way. Given the semantic coupling that exists between EXPs and NEXPs, e.g., NEXPs are expressed using "non-linear" compositions of EXPs interpreted in a context, the proposed methodology had to minimize the semantic coupling and to systematically describe the domains of the EXPs and NEXPs. The devised methodology was based on a theoretical description of the art gallery classification problem (Subsection Theoretical Description was added to the paper to describe the problem). Thus in addition to the experiments conducted to validate the two hypotheses, the devised experimental method to verify the hypotheses and the ontologies for EXPs and NEXPs for conceptual artistic photography are novel contributions of this work.

We also added the first three paragraphs in Subsection Experimental Methodology to explain how the devised methodology relates to the theoretical model of the gallery classification problem and the requirements to conduct a systematic and consistent verification of the two hypotheses.

We also redid Figure 3 to make it simpler.

Subsection Dataset Design was also modified to highlight how the experimental methodology was applied to design the datasets. Figure 5 and the second paragraph were added to clarify how the similarities/ dissimilarities of EXPs and NEXPs were tackled when building the datasets.

- *Secondary, this paper offers valuable insights into the intersection of art and artificial intelligence, raising important questions about the boundaries of artistic understanding in neural networks. This paper should do more research in the area of intricate relationship between technology and creativity, for the sake of better explanation about the significance and necessity of your research.*

Response:

The reviewer's comment was addressed as follows. The changes were highlighted in yellow in the revised document.

We rewrote the last paragraph of the section Related Work to connect it to work on creativity. We explained the limitations of technology when being used in creative activities, including art, and we explicitly stated the knowledge gap that this work attempted to address.

Thirdly, authors should present some picture about the art in your paper in order to better understand.

Response:

The reviewer's comment was addressed as follows. The changes were highlighted in yellow in the revised document.

Figure 5 and the second paragraph in subsection Dataset Design were added to clarify how the similarities/ dissimilarities of EXPs and NEXPs were tackled when building the datasets.

- *Finally, authors should share their code and project for easier reproducibility and persuasion.*

Response:

The code implementation and the information needed to reconstruct the used datasets were provided and are open-accessed via the following link on GitHub and DOI: 10.5281/zenodo.11167490.

`https://github.com/aghazahedim/How-Deep-is-Your-Art/tree/main`

Reviewer #2:

- *Based on previous comments, the manuscript has improved significantly; however, some typos and wrong sentence structure can be improved.*

Response:

We have carefully read the manuscript and hopefully corrected all typos and incorrect sentence structures.

- *Also, some improvement is needed in presenting the number of images in each of the datasets/galleries clearly and scaling them to fit the journal template guidelines.*

Response:

We have replaced Figure 4 containing datasets with a more readable version.

- *My other concern is whether the manuscript exceeds the page limits.*

Response: The text in the manuscript is actually not very long but the paper includes many figures and very big tables to explain the nature of the EXPs and NEXPs. These tables were added in response to the explicit request by the previous review iteration of the paper.

- *Overall, the manuscript can be more structured and aligned to make the paper easy to follow.*

Response:

We introduced a new Subsection on the theoretical description of the studied art gallery classification problem, and then used it to support the following subsections about the Experimental Methodology and Dataset Design.

We also moved the PCA 3D plots for Datasets S2, S3, G1, and S4 to the supplementary information part to reduce the Experiments section.

In addition to the changes that were highlighted in yellow, some sentences were re-written throughout the entire paper to improve the paper structure and presentation.
